# A Promising Anti-dengue Virus Carboxylated-Sesquiterpene Lactone from *Stevia entreriensis* (Asteraceae)

**DOI:** 10.1021/acsomega.5c05127

**Published:** 2025-10-09

**Authors:** Jimena Borgo, Alejandro Rodríguez-Martínez, Mariel S. Wagner, Augusto E. Bivona, Claudia S. Sepúlveda, César A. N. Catalán, Horacio Pérez-Sánchez, Valeria P. Sülsen

**Affiliations:** † Instituto de Química y Metabolismo del Fármaco, (IQUIMEFA) CONICET-Universidad de Buenos Aires; Cátedra de Farmacognosia, Facultad de Farmacia y Bioquímica, 28196 Universidad de Buenos Aires, C1113AAD Buenos Aires, Argentina; ‡ Structural Bioinformatics and High Performance Computing Research Group (BIO-HPC), HiTech Innovation Hub, 16728 Universidad Católica San Antonio de Murcia (UCAM), 30107 Murcia, Spain; § Health Sciences PhD Program, Universidad Católica de Murcia (UCAM), 30107 Murcia, Spain; ∥ Laboratorio de Estrategias Antivirales, Departamento de Química Biológica, Facultad de Ciencias Exactas y Naturales, Universidad de Buenos Aires, C1428EGA Buenos Aires, Argentina; ⊥ Instituto de Estudios de la Inmunidad Humoral Prof. Ricardo A. Margni (IDEHU), (Universidad de Buenos Aires- CONICET), Buenos Aires C1113AAD, Argentina; Cátedra de Inmunología, Facultad de Farmacia y Bioquímica, Universidad de Buenos Aires, C1113AAD Buenos Aires, Argentina; # Instituto de Química Biológica de la Facultad de Ciencias Exactas y Naturales (IQUIBICEN), 62873 CONICET-Universidad de Buenos Aires, C1428EGA Buenos Aires, Argentina; ∇ Instituto de Química Orgánica, Facultad de Bioquímica, Química y Farmacia, Universidad Nacional de Tucumán, T4000INI San Miguel de Tucumán, Tucumán, Argentina

## Abstract

In the ongoing search for effective antiviral agents against dengue virus (DENV), we have isolated a promising compound from a relatively unexplored natural source. This study describes the isolation, structural elucidation, and biological evaluation of a carboxylated sesquiterpene lactone, desacyl grazielia acid tiglate, from *Stevia entreriensis* (Asteraceae). Using an integrated approach that combines phytochemical and virological techniques, we reveal the potent anti-DENV activity of this compound, characterized by low cytotoxicity and high selectivity against DENV serotype 2 (EC_50_ = 2.36 μM, CC_50_ = 2527.7 μM, and SI = 1070.6). Furthermore, molecular modeling studies suggest a specific interaction mechanism with the viral NS2B/NS3 protease and NS5 polymerase, highlighting potential targets for viral inhibition. Our findings emphasize the antiviral potential of *S. entreriensis* paving the way for the development of nature-inspired therapeutics against DENV. The interdisciplinary approach reinforces the role of natural products in discovering new antiviral drugs and provides a promising candidate for further medicinal chemistry optimization.

## Graphical abstract

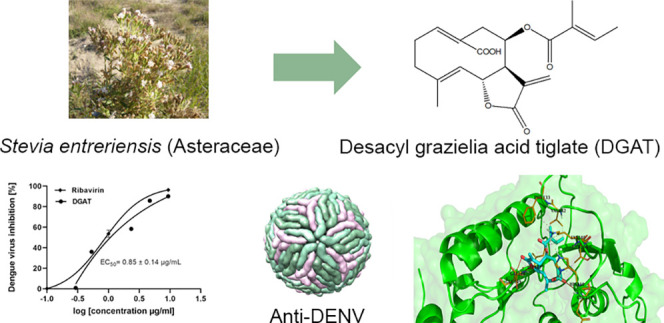

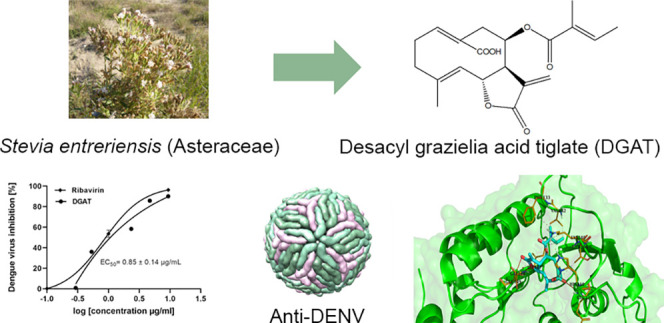

## 1 Introduction

The genus *Stevia* (tribe Eupatorieae, subfamily Asteroideae, family Asteraceae) is globally recognized for the species *Stevia rebaudiana* Bertoni (Asteraceae), popularly known as ″stevia,″ which produces large quantities of stevioside, a potent natural non-nutritive sweetener. Within the 230 species of this genus, approximately 60 species have been studied for their chemical composition.^1^ Sesquiterpene lactones (STLs) are the most characteristic secondary metabolites isolated from *Stevia* spp. STLs are 15-carbon (C15) terpenoid compounds with cyclic arrangements and numerous modifications resulting in a variety of chemical structures. These phytochemicals are compounds of moderate to low polarity and can be found superficially in the aerial parts of plants.^2^

Extensive biological activities have been attributed to STLs, including anti-inflammatory, cytotoxic, antiviral, antimalarial, antileishmanial, and trypanocidal activities, among others.^2,3^ Most of the studies have been focused on the antitumor potential of this type of compound. Several STLs have emerged as leads for cancer treatment and for suppressing metastatic progression.^4–8^ Regarding antiviral activity of STLs, it has been reported that this type of terpenoids could interfere with essential stages of the viral life cycle, including viral entry and replication. STLs can inhibit viral replication by interfering with the synthesis of viral RNA and proteins. Among STLs, guaianolides and germacranolides have shown noteworthy activity against viruses like hepatitis C, influenza, herpes simplex and SARS-CoV-2.^9–11^ On the other hand, eudesmanolides such as alantolactone and saluenolide A have shown activity against herpes simplex virus 1 and hepatitis B virus, respectively.^12^ Artemisinin has recently been pointed out as a candidate for anti-SARS-CoV-2 therapy.^13^

*Stevia entreriensis* Hieron. is a species distributed in Paraguay, southwestern and central Uruguay, and the central-eastern region of Argentina, where it grows mainly in dunes and dry or sandy soils inhabiting natural or anthropic shores and riverbanks at altitudes below 500 m above sea level.^14,15^ The roots of this species are used in infusions or decoctions in folk medicine against ″Chucho″ (intermittent fever, frequently caused by malaria) and as antidiarrheal, antidiabetic, and digestive remedies.^16^ To date, the phytochemical composition of this species is unknown. Given the reported diversity of phytochemicals from *Stevia* and their potential biological activities, its study could be promising for identifying new bioactive compounds. Moreover, the presence of sesquiterpene lactones in the genus, compounds with well-documented antimicrobial activity, further supports the relevance of investigating *S. entreriensis* as a potential source of novel antiviral agents.

Dengue fever is a viral infection classified as a Neglected Tropical Disease by the World Health Organization.^17^ It is caused by the dengue virus (DENV) (family *Flaviviridae*, genus *Orthoflavivirus*), an enveloped virus with a single-stranded positive-sense RNA genome. During the infection, DENV enters the human body through the bite of an *Aedes* sp. mosquito and primarily targets immune system cells, including dendritic cells, macrophages, monocytes, and lymphocytes.^18^ Four distinct virus serotypes (DENV-1-4), which exhibit more than 70% primary sequence homology, are distributed worldwide.^19^ DENV serotype 2 (DENV-2) has been taken as a reference for biological assays since it is considered the most virulent strain of the four serotypes.^20^ The single-stranded DENV RNA is translated into one polyprotein chain followed by cotranslational cleavage into 10 mature proteins, including three structural proteins [capsid (C), premembrane (prM), envelope (E)] and seven nonstructural proteins (NS1, NS2A, NS2B, NS3, NS4A, NS4B, and NS5). The nonstructural proteins play significant roles in evading innate immune responses, virion assembly, and genome replication. In particular, NS1, NS3, and NS5 are crucial for forming the viral particle during the infection cycle.^20^

Since half of the world’s population inhabits areas with vector circulation, 100–400 million infections occur every year.^21^ Most patients infected only experience mild illness nevertheless mortality rates of severe dengue reach 20%.^22^ No specific and effective treatments for this virus are available.^23^ Given the extensive population affected by this virus and the lack of effective therapeutics, there is an urgent need to develop new treatments against DENV.

Herein, we describe the isolation and structural elucidation of a carboxylated sesquiterpene lactone (C-STL) from the unstudied South American plant species *S. entreriensis*. We also assessed the bioactivity *in vitro* against DENV of the C-STL and its potential molecular targets *in silico*, in the search for a novel lead molecule for developing new antiviral treatments.

## 2 Results and Discussion

### 2.1 Structural Elucidation of the Isolated Compound from *S. entreriensis*

The structural elucidation of the isolated compound from *S. entreriensis* (SE) was performed by spectroscopic methods, and the experimental results were compared with those found in the literature. Compound SE was isolated as a whitish gum, soluble in CH_2_Cl_2_ and DMSO. On Thin Layer Chromatography (TLC) it showed a violet band with ANIS and a positive band with the BG reagent. It was not observable under UV light at 366 nm. According to these results, a possible acidic terpenoid was suspected. The HRESIMS was consistent with the molecular formula of C_20_H_24_O_6_ (Figure S1) (calculated for [M + Na]^+^: 383.1469; found: 383.1491; Δ 0.0022). IR spectrum is shown in Figure S2. In the ^1^H NMR spectrum of the compound (Figure S3, Table 1), signals at δ 5.65 and 6.29 were observed, corresponding to the H atoms of the exomethylene group at position 13 typically present in STLs. The doublet at δ 6.29 with *J* = 3.4 Hz (H-13a) corresponds to the vinyl hydrogen *cis* to the lactone carbonyl. Therefore, it is more deshielded than H-13b which appears at δ 5.65, also as a doublet with *J* = 3.1 Hz. Furthermore, both *J* values are greater than 3.0 Hz, indicating that the lactone ring is *trans*-fused.^15^ On the other hand, a broad singlet at δ 1.82 (3H) corresponds to the C-15 methyl group. However, the C-14 methyl group signal is missing, indicating a substitution at that position, which is unusual in STLs. The H-7 signal appeared as a double double doublet at δ 2.89 with couplings of 8.9, 3.5, and 3.0 Hz with H-6, H-13a, and H-13b, respectively. The coupling of H-7 with H-8 is negligible, indicating a dihedral angle of *ca*. 90° between them and that the ester residue at C-8 is β-oriented. In the case of H-2a (δ = 3.48) and H-9a (δ = 3.55), their chemical shifts appear at lower fields than expected due to the deshielding effect generated by the proximity to the carboxylic group, a phenomenon previously described for grazielia acid.^24^ The signals corresponding to the tiglate residue, an ester moiety commonly found in STLs, were consistent with those reported in the literature.^25^ In the ^13^C NMR spectrum of the isolated compound (Figure S4, Table 1), signals for 20 carbons were identified. The signal corresponding to the lactone carbonyl was observed at δ 169.5. Furthermore, two more carbonyl signals were evident at δ 171.3 and 166.4 that were assigned to the carbonyls of a carboxylic group and an α,β-unsaturated ester (tiglate), respectively. Eight signals for olefinic carbons were also detected between δ 151.3 and 121.1 corresponding to four C, three CH and one CH_2_. The 5-carbon side chain at C-8 corresponds to a tiglate residue.^25^ Angelate and tiglate moieties are frequently found in STLs.^26^

**Table 1:** ^1^H NMR Signals of the Isolated Compound in CDCl_3_ (600 Mz) and ^13^C NMR (125 MHz; 25° C)

position	δH (*J* in Hz)	δH reported^a^	δC, type (HSQC)	H–H COSY	HMBC
1	5.82 dd (12.9, 4.4)	5.84 br dd	151.3, CH	9a, 9b	9a, 9b
2	(a) 3.48 dddd (12.9, 12.5, 11.7, 5.4)	(a) 3.45 br dd	26.3, CH_2_	5, 3a	3b
	(b) 2.30 m	(b) 2.35 m			
3	(a) 2.48 ddd (11.7, 5.5, 1.3)	2.35 m	38.7, CH_2_	2b, 15	1, 5, 9a 15
	(b) 2.37 m			5	
4			144.5, C		3b, 15
5	4.99 dd (10.1, 0.8)	4.97 br d	125.3, CH	2b, 3b, 7	1, 2a,2b,3a, 3b, 9a, 9b, 15
6	5.12 dd (10.1, 8.9)	5.10 dd	75.6, CH	15	1
7	2.89 ddd (8.9, 3.5, 3.0)	2.89 br ddd	53.2, CH	5, 8, 9b	13a, 13b
8	5.79 d (6.4)	5.79 br d	69.5, CH	7, 9a, 9b	1′
9	(a) 3.55 dd (14.6, 6.4)	3.53 br dd	39.2, CH_2_	8, 9b	1, 2a, 5, 8, 15
	(b) 2.21 d br (14.6)	2.22 br d		1, 7, 8, 9a	
10			128.0, C		
11			136.7, C		8, 13a
12			169.5, C		13a, 13b
13	(a) 6.29 d (3.4)	6.29 d	121.1, CH_2_		
	(b) 5.65 d (3.1)	5.64 d			
14			171.3		1
15	1.82 s br (3H)	1.82 br s	17.3, CH_3_	3, 6	1, 3b, 5, 4′
1′			166.4, C		8, 14
2′			125.7, C		6, 8, 9b
3′	6.75 br q (6.8, 1H)	6.77 br q	138.2, CH	5′	
4′	1.73 br d (6.8, 3H)	1.76 br d	11.9, CH_3_		15
5′	1.74 br s (3H)	1.75 br s	14.4, CH_3_	3′	

a *J* (Hz): 1. Two = 12; 1, 2′ = 4; 2, 3 = 10; 2,2′ = 13; 2.3′ = 4; 5,6 = 6.7 = 10; 7,8 ∼ 1; 7.13 = 3.5; 7. 13′ = 3; 8.9 = 6.5; 8,9 = 14. Bohlmann et al.^27^

The compound was identified as desacyl grazielia acid tiglate (Figure 1). This lactone is the tiglate analog of the angelate described by Bohlmann et al.^24^ who named it grazielia acid.

**Figure 1:**
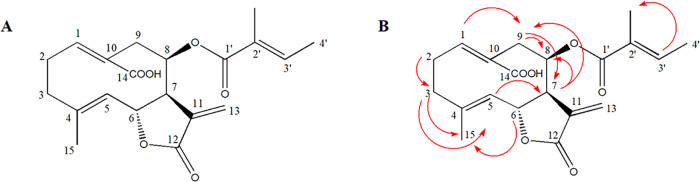
Chemical structure of desacyl grazielia acid tiglate (A). Red arrows show most relevant H–H COSY correlations (B).

The purity of desacyl grazielia acid tiglate isolated from *S. entreriensis* was determined using HPLC equipment coupled to a diode array UV–visible detector, resulting in a 96.05% purity.

Desacyl grazielia acid tiglate has been reported in three species from the Asteraceae family: *Campovassouria bupleurifolia*,^27^ *Blainvillea rhomboidei* ^28^ and *Stevia amambayensis*.^29^ A literature search revealed that no biological activities have been reported for this compound.

### 2.2 Anti-DENV-2 Activity of Desacyl Grazielia Acid Tiglate

The cytotoxicity of desacyl grazielia acid tiglate on Vero cells was evaluated after a 48 h incubation period by the MTT method. This compound showed a CC_50_ value of 0.91 ± 0.14 mg/mL (2527.7 μM). After this experiment, the antiviral activity of this compound was determined against DENV-2 at noncytotoxic concentrations by the PFU method. Ribavirin, a broad-spectrum antiviral approved for the treatment of several viral infections caused by RNA viruses but not for DENV-2, was included as a positive control for viral inhibition. The STL desacyl grazielia acid tiglate probed to be a potent inhibitor of DENV-2 with an EC_50_ of 0.85 ± 0.14 μg/mL (2.36 μM) (Figure S9) and a selectivity index (SI), calculated as the CC_50_/EC_50_ ratio of 1070.6. For ribavirin, the values were CC_50_ = 0.09 ± 0.02 mg/mL (370 μM), EC_50_ = 0.10 ± 0.01 μg/mL (0.42 μM) and SI = 900. These results demonstrate that desacyl grazielia acid tiglate has inhibitory properties against DENV-2 *in vitro* with low cytotoxicity and high selectivity toward the virus in mammalian cells. The anti-DENV activity of other STLs isolated from *Stevia* spp., namely the germacranolide santhemoidin C, isolated from *Stevia satureiifolia* var. *satureiifolia* and the guaianolide 2-oxo-8-deoxyligustrin isolated from *Stevia alpina*, have been reported recently.^30^ However, these compounds have shown moderate levels of viral inhibition compared to desacyl grazielia acid tiglate. To the best of our knowledge, this is the first time that the biological activity of a carboxylated STL has been reported. This compound seems to be less cytotoxic than expected for STLs.^31^

### 2.3 Similarity Results

In an attempt to elucidate the potential molecular mechanism of DENV inhibition exerted by desacyl grazielia acid tiglate, a database of molecules that were reported inhibitors of structural and nonstructural proteins of DENV-2 *in vitro* was constructed to compare shape, electrostatic and pharmacophoric similarities with the isolated compound. A database of 489 molecules reported as inhibitors of the proteases NS1, NS2B/NS3, NS5 and the E protein of DENV-2 was designed to compare shape similarities with desacyl grazielia acid tiglate. The 70 most similar molecules to SE were selected to compare electrostatic properties with this compound. Four molecules were found to be electrostatically similar to desacyl grazielia acid tiglate, showing EON combo values near 1.00 (Table 2) (Figure S10). Three of these compounds were reported as inhibitors of the NS5 protease of DENV-2, while one (CHEMBL1324/tolcapone) was reported to inhibit NS2B/NS3 *in vitro*. Given the shape and electrostatic similarities to desacyl grazielia acid tiglate, these proteases could represent potential targets for this compound.

**Table 2:**
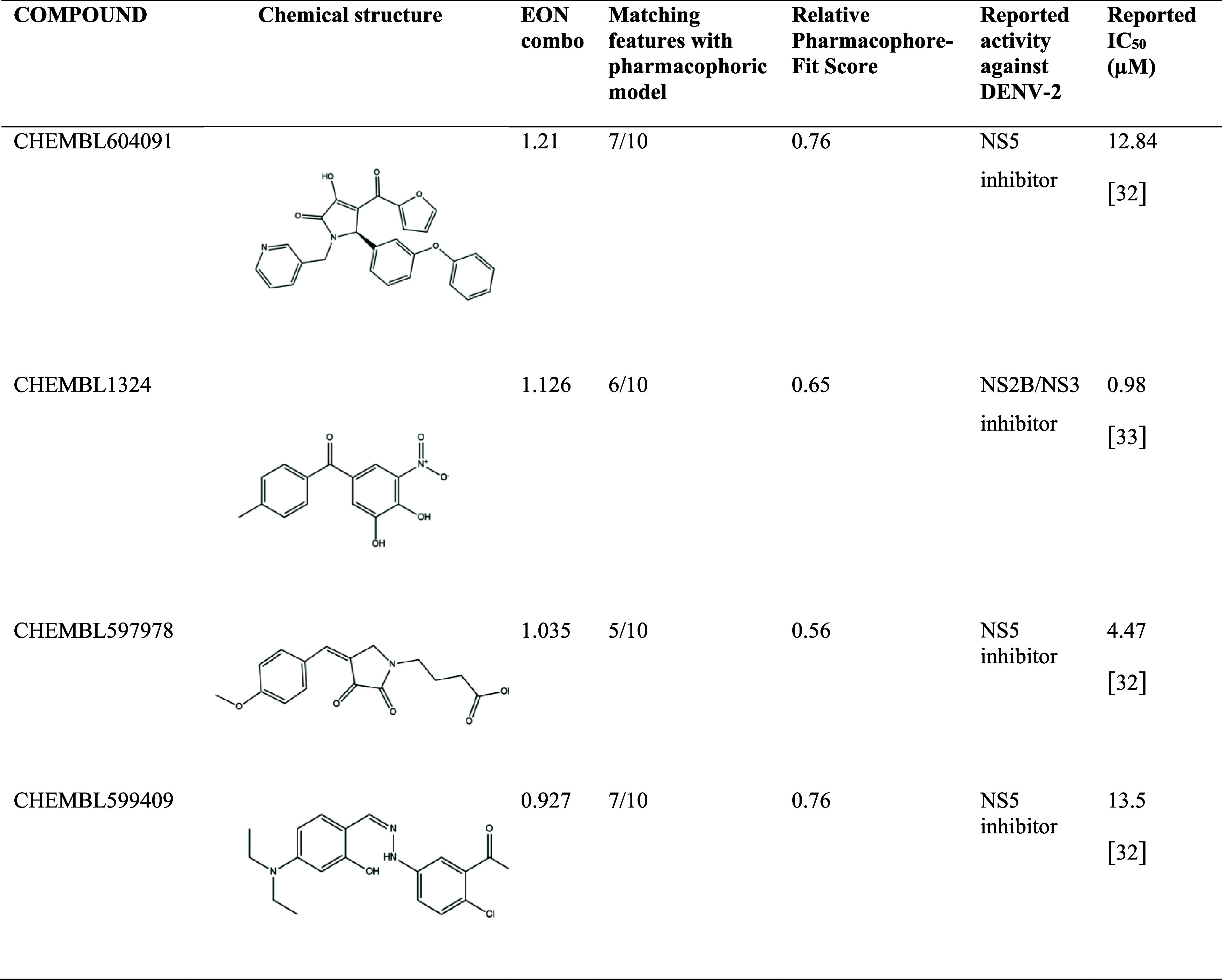
Compounds Showing the Highest Electrostatic Similarity to Desacyl Grazielia Acid Tiglate, Including Values Calculated by EON^a^ ^33^

a The EON combo score, which considers both electrostatic and Tanimoto shape similarities, is presented along with the number of matching pharmacophoric features and relative pharmacophore-fit scores obtained using the pharmacophoric model of desacyl grazielia acid tiglate. Relevant experimental data are also included.

In the analysis of pharmacophoric similarity, the top four compounds exhibited high similarity, with most pharmacophoric features matching desacyl grazielia acid tiglate pharmacophoric model aligning with those of the top four compounds. CHEMBL604091 and CHEMBL599409 demonstrated the most significant similarity, each sharing 7 out of 10 pharmacophore features, with a relative pharmacophore-fit score of 0.76. CHEMBL1324 and CHEMBL597978 displayed slightly lower matching features, with counts of 6 and 5, respectively, and corresponding pharmacophore-fit scores of 0.65 and 0.56 (Table 2, Figure S10). Three inhibitors of NS5 (CHEMBL604091, CHEMBL597978 and CHEMBL599409) and one inhibitor of NS2B/NS3 (CHEMBL1324) showed high similarity with desacyl grazielia acid tiglate.

### 2.4 Molecular Docking

In order to study the potential molecular targets of desacyl grazielia acid tiglate, blind docking simulations with every available DENV-2 target were performed. Blind docking simulations were performed between desacyl grazielia acid tiglate and the different DENV-2 molecular targets NS1, NS2B/NS3, NS5 and the E protein to identify the potential molecular mechanism of antiviral activity exerted by this compound (results shown in the Supporting Information, Table S1). The highest docking scores were observed for desacyl grazielia acid tiglate binding to NS2B/NS3 and NS5. In both cases, the best docking pose was located within the active sites described for these proteases. Consequently, focused molecular docking simulations were carried out at those active sites using desacyl grazielia acid tiglate and the CHEMBL compounds that had shown shape and electrostatic similarity to it. These docking studies aimed to evaluate and compare the binding interactions of all compounds with the target proteases.

Results showed that desacyl grazielia acid tiglate could interact with the active site of the NS2B/NS3 complex through a hydrogen bond with the residue SER135 and salt bridges with HIS51. These residues have been described as constituents of the catalytic triad of this protease, and therefore, these interactions could indicate a potential inhibition mechanism. On the other hand, the molecule CHEMBL1324 that showed shape and electrostatic similarities with desacyl grazielia acid tiglate and has been described as an inhibitor of the NS2B/NS3 protease *in vitro*, also showed overlapping interactions with the target. However, desacyl grazielia acid tiglate interacts with two catalytic triad residues, while CHEMBL1324 only interacts with HIS51 via π-stacking interactions (Figure 2, Table 3).

**Figure 2:**
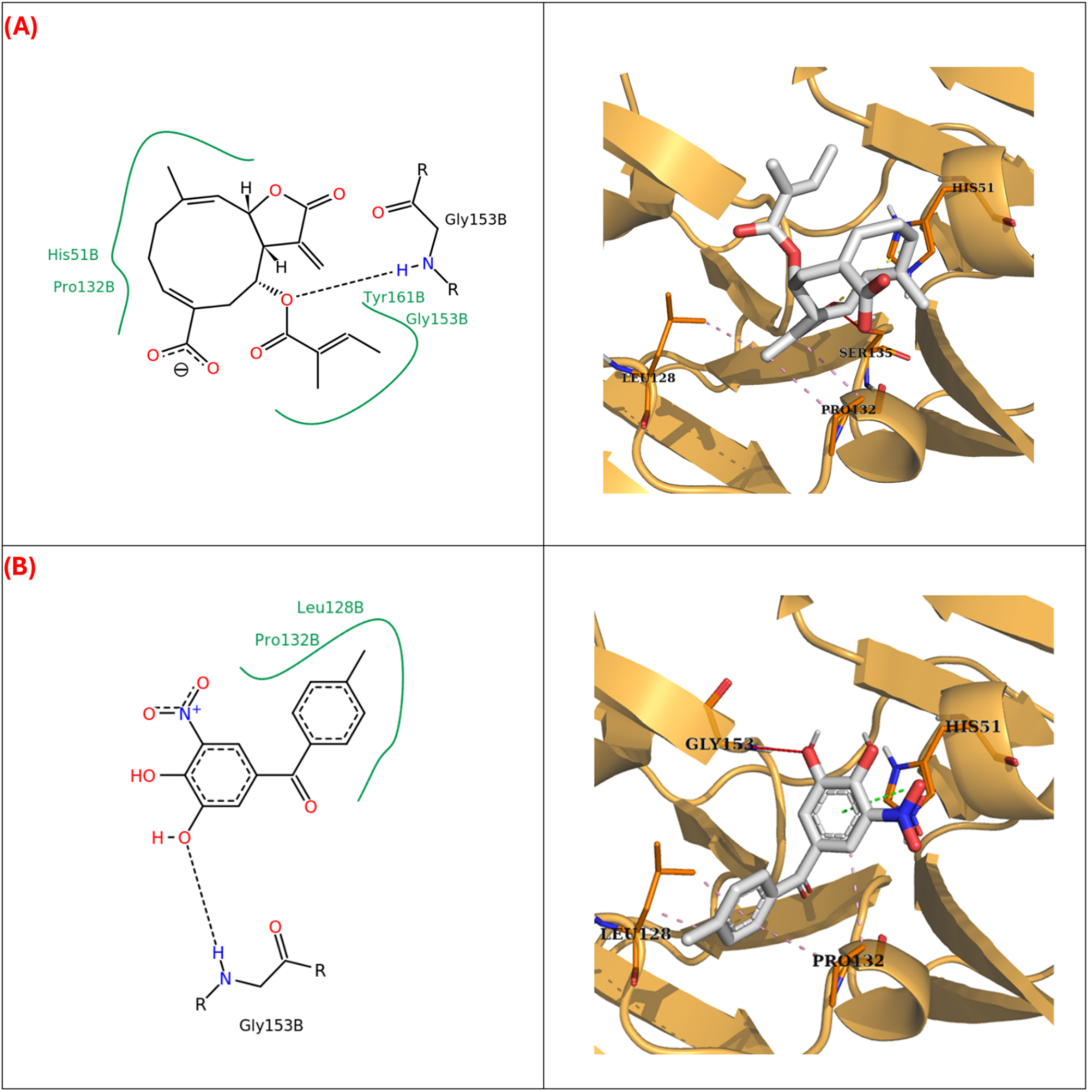
2D and 3D interaction diagrams obtained by AutoDock Vina targeted docking at the binding site of DENV-2 NS2B/NS3 with desacyl grazielia acid tiglate (A) and CHEMBL1324 (B). Pink dotted lines: Hydrophobic interactions. Yellow dotted lines: Salt bridges. Green dotted lines: π-stacking interactions. Red lines: hydrogen bonds.

**Table 3:** Docking Energies, Interactions and Distances between Desacyl Grazielia Acid Tiglate or CHEMBL1324 or CHEMBL604091 or CHEMBL597978 or CHEMBL599409 and DENV-2 Proteases NS2B/NS3 and NS5 by the Docking Method

NS2B/NS3					
ligand	docking score (kcal/mol)	hydrophobic interactions (Å)	hydrogen bonds (Å)	salt bridges (Å)	π-stacking (Å)
desacyl grazielia acid tiglate	–7.02	LEU128 (3.77)	SER135 (3.13)	HIS51 (4.90)	
		PRO132 (3.65)			
		PRO132 (3.75)			
CHEMBL1324	–8.06	LEU128 (3.99)	GLY153 (2.99)		HIS51 (4.02)
		LEU128 (3.57)			
		LEU128 (3.57)			
		PRO132 (3.87)			
		PRO132 (3.9)			

NS5					
ligand	docking score (kcal/mol)	hydrophobic interactions (Å)	hydrogen bonds (Å)	salt bridges (Å)	π-stacking (Å)
desacyl grazielia acid tiglate	–8.18	THR104 (3.69)	GLU111 (3.09)	LYS105 (5.42)	
		LYS105 (3.69)	GLY148 (3.06)	HIS110 (4.27)	
		VAL132 (3.46)			
		VAL132 (3.7)			
		PHE133 (3.7)			
		ILE147 (3.65)			
		GLU149 (3.73)			
CHEMBL604091	–8.76	THR104 (3.74)	CYS82 (4.08)		
		LYS105 (3.83)	GLY148 (3.17)		
		LYS105 (3.52)	GLU149 (3.78)		
		LYS105 (3.72)			
		VAL132 (3.96)			
		VAL132 (3.43)			
		PHE133 (3.7)			
		PHE133 (3.64)			
		ILE147 (3.68)			
CHEMBL597978	–6.74	LYS105 (3.86)	GLY86 (3.34)		
		LYS105 (3.9)	TRP87 (3.6)		
		ILE147 (3.73)	ASP146 (3.72)		
		ILE147 (3.96)			
CHEMBL599409	–6.42	THR104 (3.74)	GLY83 (3.63)		
		LYS105 (3.56)	VAL130 (2.72)		
		LYS105 (3.69)	VAL132 (3.68)		
		GLU111 (3.72)			

Regarding the docking simulations between desacyl grazielia acid tiglate and the protease NS5, the binding site is in accordance with the active site and hydrophobic interactions with the residue VAL132, which is essential for NS5 enzymatic activity, are present. Additionally, the molecule CHEMBL604091 displayed similar interactions and docking scores to desacyl grazielia acid tiglate, while molecules CHEMBL597978 and CHEMBL599409 showed less interactions and docking scores than SE. These compounds were selected because of their shape and electrostatic similarities with compound SE and since they are reported as inhibitors of DENV-2 NS5 *in vitro*, these results could indicate that the antiviral properties exerted by desacyl grazielia acid tiglate could be similar to those of molecule CHEMBL604091 through the inhibition of NS5 (Figure 3, Table 3).

**Figure 3:**
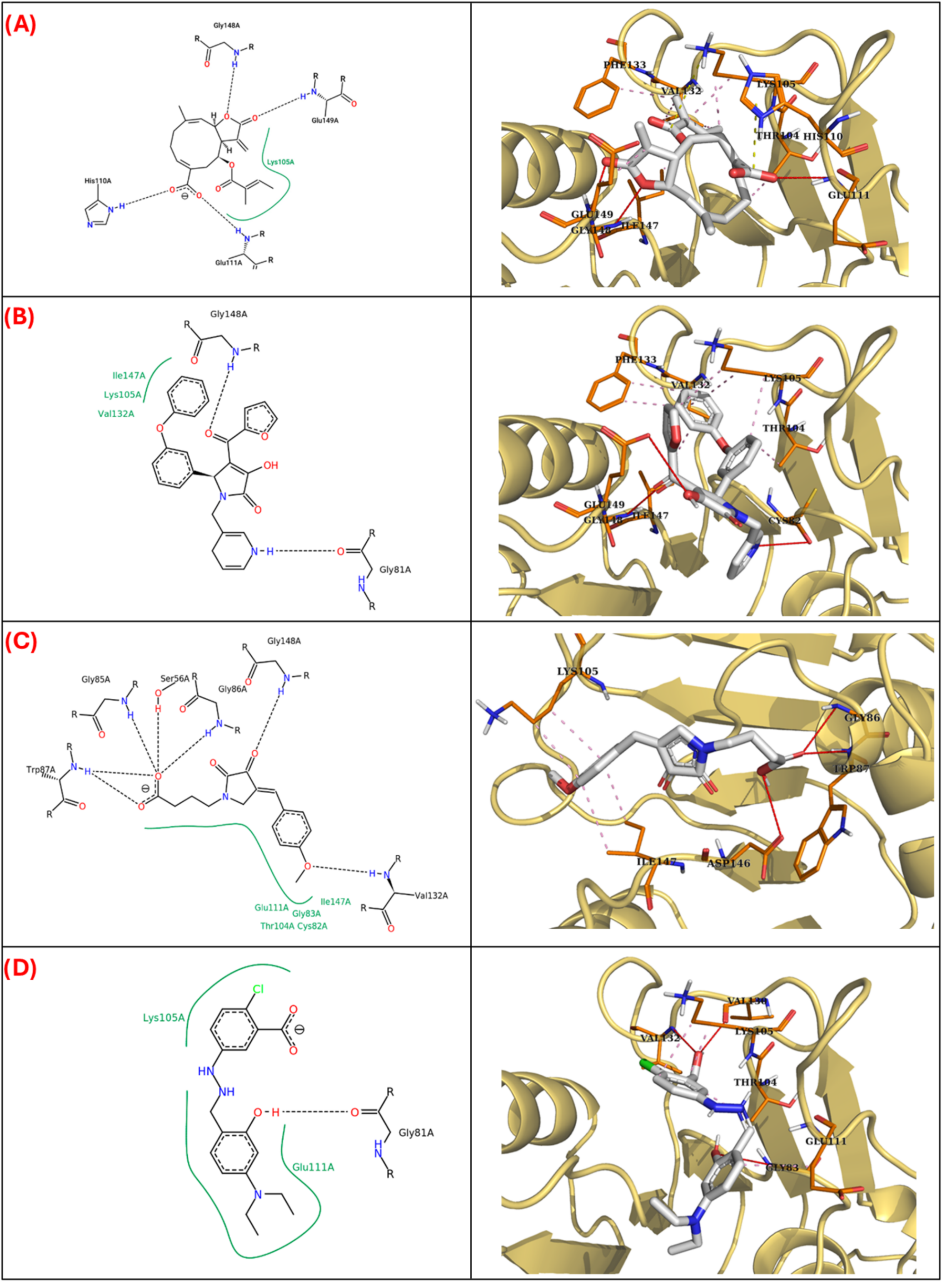
2D and 3D interaction diagrams obtained by AutoDock Vina targeted docking in the binding site between DENV-2 NS5 and desacyl grazielia acid tiglate (A), CHEMBL604091 (B), CHEMBL597978 (C), and CHEMBL599409 (D). Pink dotted lines: Hydrophobic interactions. Yellow dotted lines: Salt bridges. Red lines: Hydrogen bonds.

The NS3 protein of DENV displays protease, helicase and RNA triphosphatase activities. This protein binds to another nonstructural DENV protein, NS2B, which acts as a cofactor by shielding hydrophobic residues of NS3. The NS2B/NS3 complex has been considered a promising target protein for potential antiviral molecules, since its inhibition has been associated with the interruption of viral replication and decreased infectivity. A highly conserved catalytic triad (HIS51-ASP75-SER135) present in NS3 is vital for its activity, and therefore, it constitutes a binding site for inhibitors.^34,35^ On the other hand, NS5 presents dual enzymatic activity, with a methyl-transferase domain in its N-terminus, which catalyzes the post-transcriptional process of RNA capping, and an RNA-dependent RNA polymerase domain located at its C-terminus, responsible for the replication of the positive-strand RNA in the host cell. Interactions with residues VAL132 and ASP131 have been reported as desirable for inhibitors of this DENV protein. Due to its critical biological role, NS5 emerges as a prime target for therapeutic interventions against the DENV.^32,36–38^

Overall, results showed that desacyl grazielia acid tiglate had similar interactions to NS2B/NS3 than the inhibitor CHEMBL1324 and interacted with two residues (SER135 and HIS51) within the catalytic triad of this protease. At the same time, CHEMBL1324 interacted with one of these residues (HIS51).

### 2.5 Molecular Dynamics Simulations

A MD simulation for each complex was performed to check the interactions and stability of the protein–ligand binding. We calculated the MM-PBSA (Molecular Mechanics Poisson–Boltzmann Surface Area) plots to show the stability of each ligand in the corresponding protease. Later, we computed the interaction energy values for the nonbonded interactions between the ligand and the protein for each case. Figure 4 depicts the binding energy values obtained by the MM-PBSA method during all the MD simulations. This graph allows us to examine the stability of the protein–ligand complex in order to check if the compounds could be potential ligands for the respective protein. Thus, the NS2B/NS3- desacyl grazielia acid tiglate complex stabilizes between −150 and −200 kJ/mol, staying stable for most of the simulation time. The complex formed by NS2B/NS3 and CHEMBL1324 shows greater instability during the simulation. Hence, this stabilization difference between the two complexes can be positive evidence for describing desacyl grazielia acid tiglate as a possible ligand or inhibitor of the NS2B/NS3 protease.

**Figure 4:**
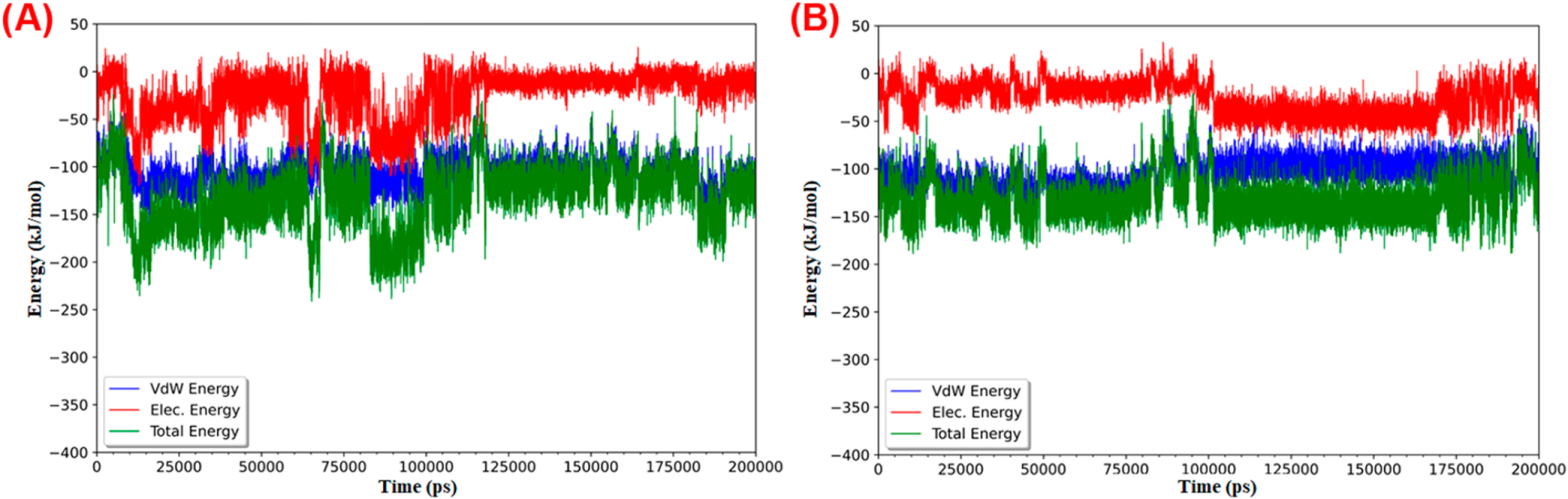
MM-PBSA representations of NS2B/NS3 complexes with different compounds: (A) NS2B/NS3- desacyl grazielia acid tiglate complex and (B) NS2B/NS3-CHEMBL1324 complex.

The results for the NS5- desacyl grazielia acid tiglate complex (Figure 5) demonstrate the stability of the total energy, maintaining approximately −200 kJ/mol from 30,000 ps onward. This stabilization suggests that desacyl grazielia acid tiglate enhances the stability of the protease through effective binding and complex formation with the protein. Similarly, the reference compounds exhibited stabilization at binding energy levels around −200 kJ/mol during the final stages of the simulation. These findings support the hypothesis that desacyl grazielia acid tiglate acts as a ligand, capable of stabilizing the NS5 protein in a manner comparable to the three reference molecules.

**Figure 5:**
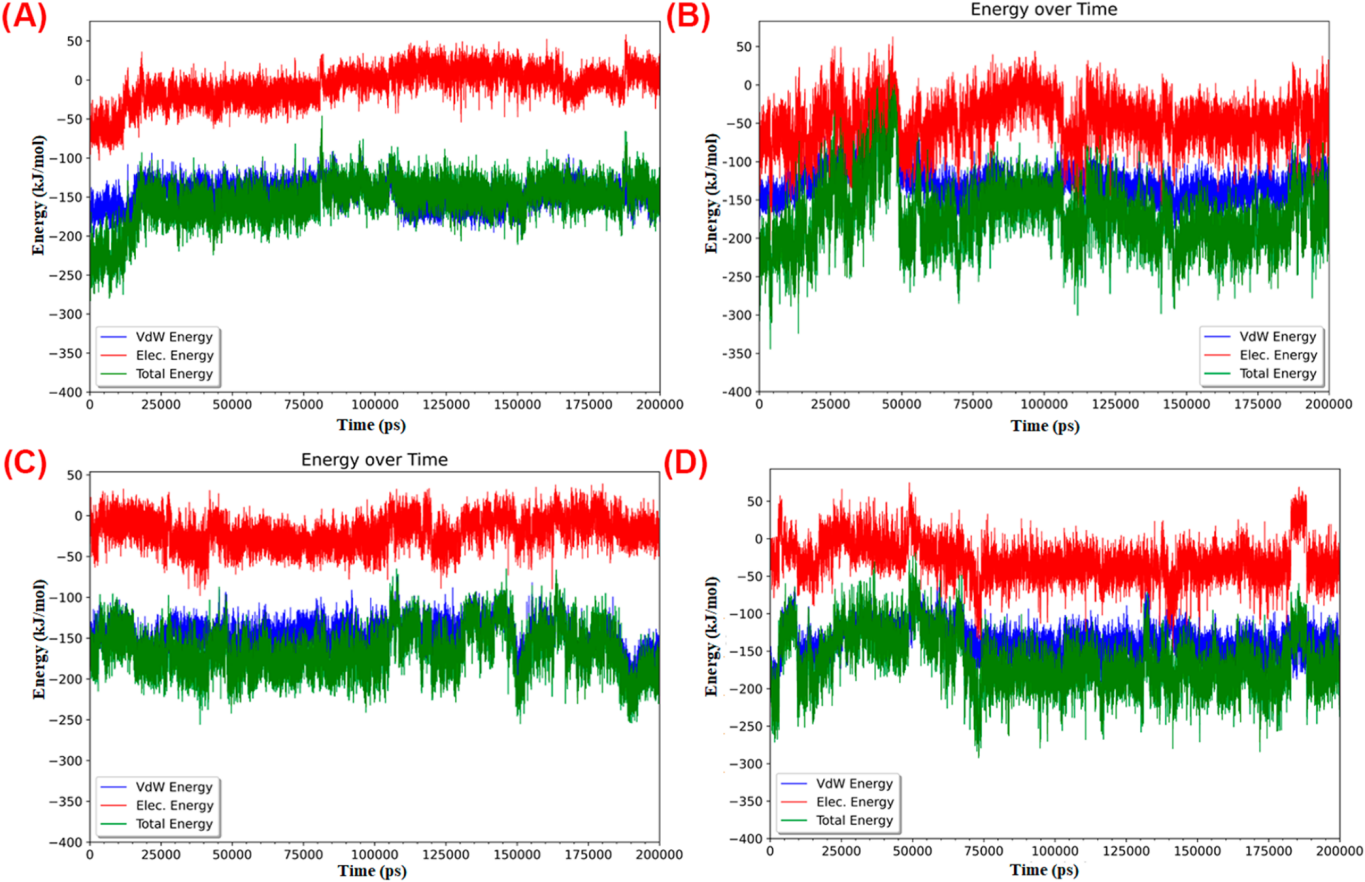
MM-PBSA representations of NS5 complexes with different compounds: (A) NS5- desacyl grazielia acid tiglate complex, (B) NS5-CHEMBL597978 complex, (C) NS5-CHEMBL599409 complex, and (D) NS5-CHEMBL604091 complex.

Finally, the results obtained from the various analyses are based on molecular dynamics simulations, including RMSD, RMSD fluctuation, radius of gyration (Rg), and hydrogen bond interaction analysis. These analyses are consistent with the binding mode of these molecules to the NS2B/NS3 and NS5 proteins. These findings support and validate the information previously obtained from the molecular docking studies (Figures S12–S23).

Regarding interactions detected, the MD simulations show nonbonded interactions in some of the critical residues already studied in the docking calculations. For the NS2B/NS3 complexes, there are interactions in key residues obtained by docking and described in the literature^19^ in both the complex formed by desacyl grazielia acid tiglate and the CHEMBL1324 inhibitor. One reported residue was HIS51, with an energy value for desacyl grazielia acid tiglate higher than that of CHEMBL1324 complex. The interaction with SER135 is also of higher value in the complex formed by desacyl grazielia acid tiglate compared to the NS2B/NS3-CHEMBL1324 complex. Other coinciding interactions between the two compounds in docking calculations are also found in the MD simulations results, as with LEU128 and PRO132 residues (Table 4).

**Table 4:** Non-Bonded Interactions between Desacyl Grazielia Acid Tiglate and CHEMBL1324, CHEMBL604091, CHEMBL597978 and CHEMBL599409 Compounds, with DENV-2 NS2B/NS3 and NS5 Proteases Residues Calculated by MD Simulations

NS2B/NS3			NS5				
residue	desacyl grazielia acid tiglate	CHEMBL1324	Residue	desacyl grazielia acid tiglate	CHEMBL604091	CHEMBL597978	CHEMBL599409
MET49	–1.2 ± 0.4		LEU80	–1.3 ± 0.7		–7.2 ± 4.4	
HIS51	–17.7 ± 9.3	–4.4 ± 9.6	GLY81	–4.2 ± 3.3	–4.7 ± 8.9	–8.2 ± 6.5	–2.4 ± 2.4
ARG54	–4.5 ± 5.1		CYS82	–1.4 ± 1.1	–1.5 ± 3.1	–1.3 ± 1.8	
ASP75	–3 ± 2.8		GLY83	–1.7 ± 2.1	–2.3 ± 5.2		
LEU128	–14 ± 4.8	–15.7 ± 9.0	ARG84		–1.1 ± 2.9		
ASP129		–3.5 ± 5.2	THR104	–7.9 ± 2.6		–10.5 ± 6.6	
PHE130	–1.1 ± 1.5	–10.1 ± 10.3	LYS105	–16.6 ± 6.0	–6.1 ± 11.5	–14.7 ± 10.2	
SER131	–2 ± 2.0	–5.9 ± 6.8	HIS110	–14.9 ± 5.0	–9.1 ± 15.2	–6.4 ± 6.0	–21.3 ± 8.9
PRO132	–5 ± 4.7	–11.2 ± 7.6	GLU111	–8.4 ± 3.9	–2.8 ± 5.9		–8 ± 5.4
GLY133		–1.3 ± 1.7	GLU112	–1.7 ± 1.0			
THR134	–1.1 ± 1.6	–2.4 ± 3.5	LEU103	–2.1 ± 1.2		–3.4 ± 3.7	
SER135	–6.1 ± 4.2	–3.1 ± 4.0	THR104		–2.8 ± 5.8		
TYR150	–4.6 ± 2.4	–6.1 ± 7.0	GLY106	–12.4 ± 6.5	–1.5 ± 3.5		
GLY151	–14 ± 5.1	–5 ± 4.6	GLY107	–5.4 ± 4.0			
ASN152	–10.2 ± 5.9	–1.9 ± 1.6	PRO108		–1.1 ± 3.3		
GLY153	–11 ± 5.8	–4.6 ± 3.5	GLY109				–6.1 ± 3.5
VAL154		–1.1 ± 1.7	VAL130	–2.4 ± 1.3		–4.3 ± 3.9	
VAL155		–1.8 ± 3.7	ASP131	–5.8 ± 4.0	–2.3 ± 4.6	–4.9 ± 6.0	–1.9 ± 1.2
TYR161	–42.2 ± 12.1	–18.7 ± 13.6	VAL132	–4.1 ± 1.8	–1.2 ± 2.6	–10.4 ± 6.1	–2.8 ± 1.2
SER163	–7.1 ± 6.8		PHE133		–1.7 ± 3.1	–8.7 ± 3.4	–4.9 ± 1.5
			ASP146	–3.1 ± 4.1	–2.3 ± 5.0	–1.5 ± 3.9	–5.7 ± 3.3
			ILE147	–17.7 ± 10.5	–5.6 ± 8.6	–13.4 ± 4.0	–29.2 ± 5.5
			GLY148	–10.8 ± 10.7	–3.8 ± 6.7	–5.8 ± 4.4	–24.2 ± 6.1
			GLU149	–11.6 ± 6.1	–3.6 ± 8.6	–18.4 ± 15.0	–21.9 ± 7.0
			SER150			–1.7 ± 4.8	–2.4 ± 2.2
			SER151			–1.5 ± 4.1	
			ARG160	–16.3 ± 4.3	–5 ± 12.7	–16.6 ± 10.6	–13.6 ± 6.8
			THR161			–1.3 ± 0.9	–2.1 ± 0.7
			ARG163	–18.4 ± 11.8	–1.2 ± 5.1	–18.5 ± 14.7	–19.2 ± 5.9
			VAL164	–1.1 ± 0.7		–6.4 ± 3.6	–9.1 ± 2.9
			LEU167			–4.2 ± 3.1	–4.9 ± 1.6
			LYS181		–1.1 ± 3.0		
			LEU183				–1.7 ± 0.9
			GLU217				–1 ± 0.7
**total**	–144.8 ± 74.4	–96.7 ± 93.6	**total**	–169.2 ± 92.8	–60.9 ± 125.4	–169.4 ± 127.5	–182.4 ± 67.6

The MD simulations of the four complexes with NS5 revealed results that align with those obtained from docking calculations. Critical residues previously identified in the literature^19^ and by other *in silico* methods were also observed participating in nonbonded interactions in the MD simulation outcomes. Notably, VAL132 was detected in all four complexes. Furthermore, significant interactions involving LYS105, ILE147, GLY148, and GLU149 were identified in both the complex with the SE compound and those with known inhibitors (Table 4).

Finally, results showed that desacyl grazielia acid tiglate has similar interactions with NS2B/NS3 than the inhibitor CHEMBL1324 and the interaction with two residues (SER135 and HIS51) within the catalytic triad of this protease. At the same time, CHEMBL1324 interacted with one of these residues (HIS51). This information was in accordance with the results of the MD simulations that showed that the NS2B/NS3- desacyl grazielia acid tiglate complex had greater stability during the simulation compared with the NS2B/NS3-CHEMBL1324 complex. Regarding the site-directed molecular docking of NS5, desacyl grazielia acid tiglate presented similar interactions to CHEMBL604091, a reported *in vitro* inhibitor of NS5. Both molecules interacted with the residue VAL132, which is essential for the activity of this protease. In the MD calculations, the complexes between the target, desacyl grazielia acid tiglate and the inhibitors from the database, showed stabilization at the same binding energy levels and similar interactions than those observed in the docking calculations. Given the similarity between the docking and the MD results, we hypothesized that the isolated compound from *S. entreriensis* desacyl grazielia acid tiglate could execute the activity of DENV-2 inhibitor by targeting the NS2B/NS3 and NS5 proteins.

## 3 Conclusions

In this study, we isolated and identified the C-STL desacyl grazielia acid tiglate from *S. entreriensis* as a potent antiviral compound against DENV-2, highlighting the plant’s untapped potential for yielding bioactive molecules. Demonstrating low cytotoxicity and high specificity to DENV-2, this compound presents a promising avenue for therapeutic application. Our molecular modeling analysis suggests the NS2B/NS3 protease and NS5 polymerase as potential targets, indicating a mechanism that could effectively inhibit the viral life cycle. These findings underscore the significance of natural products in discovering new antiviral agents and set the stage for further exploration of *S. entreriensis*.

Overall, the identification of desacyl grazielia acid tiglate as an anti-DENV agent opens new paths for developing dengue therapeutics, emphasizing the critical role of plant-derived compounds in the fight against viral diseases. This study paves the way for further investigations into natural resources for antiviral drug discovery, offering hope for new treatments.

## 4 Experimental Procedures

### 4.1 Plant Material

Wild specimens of native Argentinian plant species *S. entreriensis* Hieron. (Asteraceae) located in Entre Ríos Province, Médanos, Islas del Ibicuy Department, Argentina (S 33° 25.238′ W 59° 06.106′) were collected in February 2022. The harvested material was obtained by cutting 10–15% of each plant to preserve the genetic resource. The collected material was identified by renowned botanist Hernán Bach afterward. A voucher specimen was deposited at the Museo de Farmacobotánica “Juan A. Domínguez” at Facultad de Farmacia y Bioquímica, Universidad de Buenos Aires under the identification code BAF16116.

### 4.2 Plant Extract Obtention

The aerial parts of *S. entreriensis* (575 g) were dried at room temperature and protected from sunlight to preserve the natural products this species contains. Once dried, the plant material was manually grounded and extracted by maceration with dichloromethane (DCM) (Sintorgan, Buenos Aires, Argentina) for 5 min at a 10% w/v concentration. The extraction process was repeated twice, and the resulting extracts were gathered and filtered (grade 0859, medium, smooth, 90 mm, Schleicher and Schuell—Whatman, Maidstone, UK). The DCM extract was taken to dryness under reduced pressure at 40 °C in a rotary evaporator to obtain the organic crude extract.

### 4.3 Extract Processing

The dried crude extract was submitted to a dewaxing protocol in order to eliminate sterols, triterpenes, carotenoids, and waxes and to enrich the extract in medium polarity compounds like sesquiterpenes and diterpenes. Consequently, the extract (12 g) was resuspended in 140 mL of ethanol (ETOH) (Sintorgan, Buenos Aires, Argentina) in a water bath at 40 °C and diluted with distilled water (H_2_O) to a solvent ratio of ETOH:H_2_0 (70:30). The hydroalcoholic suspension was extracted first with 50 mL of hexane (HX) (3×) (Sintorgan, Buenos Aires, Argentina), and then with 50 mL of DCM (3×). The resulting DCM extracts were gathered, mixed with sodium sulfate as a drying agent, filtered and taken to dryness under vacuum to afford the dewaxed extract (4.8 g).

### 4.4 Fractionation of the Crude Extract

The semipurified dewaxed extract (4.8 g) was fractionated by column chromatography (30 × 2.5 cm) with Silica gel 60 as the stationary phase (100 g, 0.063–0.2 mm, 70–230 mesh ASTM, Macherey-Nagel, Düren, Germany). Once the extract was loaded onto the column, the elution was performed with a gradient of dichloromethane:methanol (DCM:MEOH) of 100:0 to 80:20. Forty fractions were collected and analyzed by TLC using silica gel F_254_ (Merck, Darmstadt, Germany) as the stationary phase (SP), hexane:ethyl acetate (HX:ETAC) (5:5) as the mobile phase (MP), and sulfuric anisaldehyde (ANIS) as the spraying reagent (SR). The TLC analysis of fraction 13, which was eluted with DCM:MEOH (9:1), revealed the presence of a major compound. This fraction was taken to dryness under vacuum, after which a gum soluble in DCM was obtained. TLC analysis of fraction 13 was performed with SP: silica gel F_254_, MP: DCM: MEOH (9:0.5) and SR: bromocresol green (BG). This reagent can indicate the presence of carboxylic acids, which are observed as yellow bands on a blue-colored background plate. An intense yellow band was detected in the fraction. Therefore, a purification process based on the acidic properties of the compound was designed.

### 4.5 Purification of Fraction 13

Fraction 13 (1.5 g) was dissolved in DCM (30 mL) and extracted three times with 8% w/v NaHCO_3_. The alkaline phases were combined and acidified dropwise with 85% formic acid until the pH turned acidic, as indicated by litmus paper. Once the aqueous phase was acidified, it was extracted three times with DCM. The DCM phases were combined and evaporated to dryness using a rotary evaporator to give a gummy residue. Subsequently, the gummy residue was purified using a chromatographic column (8 × 1 cm) packed with 20 g of Silica gel 60 (230–400 mesh) and eluted isocratically with HX:ETAC (3:7). Ten fractions of 4 mL each, named F13a-F13j, were collected. Fractions F13c and F13d revealed the presence of a pure compound (19 mg). Therefore, these fractions were combined and evaporated to dryness. This compound was named compound SE. The purity of compound SE was determined using high-performance liquid chromatography (HPLC). A Waters chromatograph equipped with a diode array UV–visible detector (Waters 2996) and a Waters Delta 600 pump was employed. An Agilent Eclipse Plus C-18 analytical column of 4.6 × 250 mm and 5 μm particle size was utilized. The compound SE was dissolved in acetonitrile: H_2_O (1:1) at a 0.5 mg/mL concentration. Subsequently, the prepared solution was filtered using 0.45 μm nylon filters (Agilent). The sample was eluted with a gradient of water (A) and acetonitrile (B) from 35% B to 95% B in 30 min. A 20 μL loop and a 1 mL/min flow rate were used. The UV detector was set at 210 nm. A blank run was performed by injecting the solvent mixture used to solubilize the compounds. The purity of the compound SE (%) was calculated as (100 x peak area of the compound)/∑ of every peak.

### 4.6 Identification of the Isolated Compound

The identity of the compound SE as desacyl grazielia acid tiglate was determined by IR, proton nuclear magnetic resonance (^1^H NMR) and carbon nuclear magnetic resonance (^13^C NMR), heteronuclear single quantum correlation (HSQC), heteronuclear multiple bond correlation (HMBC), ^1^H–^1^H correlated spectroscopy (COSY) (Bruker Avance 600) (600 MHz in CDCl_3_), and high-resolution electrospray ionization-mass spectrometry (HRESIMS) (Bruker micrOTOF-Q II) by comparing the experimental spectra with data from the literature.

^1^H NMR (600 MHz, CDCl_3_): δ (ppm) 6.75 (br q, *J* = 6.8 Hz, 1H), 6.29 (d, *J* = 3.4 Hz, 1H), 5.82 (dd, *J* = 12.9, 4.4 Hz, 1H), 5.79 (d, *J* = 6.4 Hz, 1H), 5.65 (d, *J* = 3.1 Hz, 1H), 5.12 (dd, *J* = 10.1, 8.9 Hz, 1H), 4.99 (dd br, *J* = 10.1, 0.8 Hz, 1H), 3.55 (dd, *J* = 14.6, 6.4 Hz, 1H), 3.48 (dddd, *J* = 12.9, 12.5, 11.7, 5.4 Hz, 1H), 2.89 (ddd, *J* = 8.9, 3.5, 3.0 Hz, 1H), 2.48 (ddd, *J* = 11.7, 5.5, 1.3 Hz, 1H), 2.37 (m, 1H), 2.30 (m, 1H), 2.21 (d br, *J* = 14.6 Hz, 1H), 1.82 (s br, 3H), 1.74 (s br, 3H), 1.73 (d br, *J* = 6.8, 3H).

^13^C NMR (151 MHz, CDCl_3_): δ (ppm) 171.3, 169.5, 166.4, 151.3, 144.5, 138.8, 136.7, 128.0, 125.7, 125.3, 121.1, 75.6, 69.5, 53.2, 39.2, 38.7, 26.3, 17.3, 14.4, 11.9.

IR (cm-1): 3392.24, 2929.93, 1752.21, 1706.23, 1648.21, 1439.28, 951.37, 896.79 cm^–1^.

### 4.7 Cells and Virus

Vero cells (derived from African green monkey kidney, ATCC CCL-81) were cultured in Eagle’s minimum essential medium (MEM, Gibco Thermo Fisher Scientific, Waltham, MA) supplemented with 5% heat-inactivated newborn bovine serum (NBS, Gibco Thermo Fisher Scientific, Carlsbad, CA) and 1.5 g/L of sodium bicarbonate under a 5% CO_2_ atmosphere. The serum concentration was reduced to 1.5% for the maintenance medium (MM). The C6/36 HT mosquito cell line, derived from *Aedes albopictus* (ATCC CRL-1660) and adapted to grow at 33 °C, was cultured in L-15 Medium (Leibovitz) supplemented with 0.3% tryptose phosphate broth, 0.02% glutamine, 1% MEM nonessential amino acids solution, and 5% FBS. The original stock of DENV-2 NGC strain was propagated in C6/36 cells and quantified via plaque formation in Vero cells.

### 4.8 Cytotoxicity Assay

Confluent monolayers of Vero cells were exposed for 48 h to serial 2-fold dilutions of the pure compound in MM, in 96-well plates. Desacyl grazielia acid tiglate was evaluated at concentrations between 0.005 and 0.15 mg/mL, and each concentration was tested in four wells to assess viability. The compound was dissolved in DMSO and diluted as necessary for the assay. The final concentration of DMSO in the wells did not exceed 0.01% DMSO. Control experiments involved treating cell cultures with a 0.01% concentration of the vehicle (DMSO). Cell viability was determined using the 3-(4,5-dimethylthiazol-2-yl)-2,5-diphenyl tetrazolium bromide (MTT, Sigma-Aldrich, St. Louis, MO) assay by adding 10 μL of MM containing MTT reagent (final concentration of 0.5 mg/mL) to each well. Following a 2-h incubation period at 37 °C, the supernatants were removed, and 100 μL of ethanol was added per well to dissolve the formazan crystals. The absorbance of the samples was measured at 595 nm. Viability values (% relative to the cell control) were determined, and the cytotoxic concentration 50% (CC_50_) was calculated using nonlinear regression analysis with GraphPad Prism (v9.0) software. CC_50_ represents the compound concentration necessary to reduce the MTT signal by 50% compared to cell controls treated with the vehicle (DMSO) and is presented as the mean ± standard deviation (SD) derived from three independent experiments.

### 4.9 Antiviral Assay

Antiviral activity was assessed through a virus yield inhibition assay. Cells cultured in 24-well microplates were infected with DENV-2 at a multiplicity of infection (MOI) of 0.1 PFU/cell. After a 1-h adsorption period at 37 °C, the unadsorbed virus was removed by washing with PBS, and cells were replenished with MM containing 2-fold dilutions of each desacyl grazielia acid tiglate concentration or the vehicle (DMSO). Ribavirin (Sigma-Aldrich, St. Louis, MO), a guanosine analogue with broad antiviral activity, was used as a positive control for DENV-2 inhibition.^39^ The concentration range used to assess antiviral activity was determined by the CC_50_ calculated for desacyl grazielia acid tiglate and ribavirin. A negative control (0.01% DMSO) was included. Following 48 h of infection, supernatants were harvested to prepare serial dilutions that were used to infect Vero cell monolayers to quantify virus yield using the plaque-forming unit (PFU) method. After a 1-h adsorption period at 37 °C, the unadsorbed virus was removed, MM supplemented with 0.7% methylcellulose was added, and cells were incubated for 1 week at 37 °C under a 5% CO_2_ atmosphere. Subsequently, cell monolayers were fixed with 10% formaldehyde, stained with a 1% crystal violet solution, and PFUs were counted. Inhibition values (% relative to viral control) were determined, and the effective concentration 50% (EC_50_), representing the concentration required to reduce virus yield by 50% in compound-treated cultures, was calculated using nonlinear regression analysis with GraphPad Prism (v9.0) software. Results were compared with the viral control treated with the vehicle (DMSO) and presented as the mean ± standard deviation (SD) from three independent experiments.

### 4.10 Molecular Similarity Studies

To study the potential mechanism of DENV-2 inhibition displayed by the isolated compound from *S. entreriensis*, a database of molecules that were reported to inhibit DENV-2 was constructed to compare electrostatic and shape similarities with the compound SE. In this sense, the ChEMBL^40^ and the BindingDB^41^ databases were used to search for molecules that inhibited the different molecular targets reported for DENV-2, including NS1, NS2B/NS3, NS5, and the E protein. A total of 489 compounds met the search criteria. The SMI of these molecules was obtained to be transformed into SDF files using the Open Babel tool.^42^ Afterward, the SDF files were transformed to PDBQT format with the Autodock Tools software.^43^

Once the molecules from the database were prepared as aforementioned, the OpenEye software was executed to perform a shape comparison with the ROCS toolkit (OpenEye Scientific Software, Santa Fe, N.M., U.S.) within the software in order to find the compounds from the database that resulted most similar to the isolated compound SE. The molecules of the database were listed based on the higher shape similarity to compound SE by the Tanimoto_shape score. The first 70 molecules were selected to evaluate their electrostatic similarities with desacyl grazielia acid tiglate. Consequently, the chosen molecules were analyzed with the toolkit EON (OpenEye Scientific Software, Santa Fe, N.M., U.S.) of the OpenEye software to compare the compound SE with the database. The results were sorted by the higher EON_Et_Combo score as a measure of electrostatic similarity with desacyl grazielia acid tiglate.

As a complementary technique to check the similarity with known DENV-2 inhibitors, we launched a pharmacophoric similarity screening using the Metascreener software (https://github.com/bio-hpc/metascreener) with LigandScout software^44^ as selected virtual screening engine. Thus, we created an LDB format library with the 70 first molecules selected by ROCS results and generated the pharmacophore model in PMZ format from the SDF file of the SE compound. Afterward, we ran a pharmacophoric similarity screening indicating 4 as the maximum number of omitted features for the SE compound model against the LDB library generated.

### 4.11 Molecular Simulation Studies

Molecular docking simulations were conducted to gain mechanistic insights into the structure and primary interactions between DENV-2 proteases, the E protein, and desacyl grazielia acid tiglate.

The structural conformations for DENV-2 proteases NS1, NS2B/NS3, NS5 and the E protein were obtained from the X-ray crystal structure in the PDB Database (PDB ID: 4O6B,^45^ 2FOM,^46^ 1R6A^47^ and 1OKE,^48^ respectively). Subsequently, the structure files were processed with the Autodock Tools software^43^ and converted to a PDBQT format using default parameters.

Regarding the preparation of the ligands SE, CHEMBL604091, CHEMBL1324, CHEMBL597978, and CHEMBL599409, the SMI files were converted to SDF format with the Open Babel converter tool.^42^ Afterward, the SDF files were transformed to PDBQT files with Autodock Tools,^43^ while partial charges were assigned using Gasteiger model.^49^

### 4.12 Molecular Docking

Once the ligand SE and protein structures were prepared, molecular docking calculations based on the Blind Docking (BD) technique^50^ were carried out for each proposed DENV-2 target using the Blind Docking Server (BDS, available at http://bio-hpc.ucam.edu/achilles) with Autodock 4 ^43^ as the docking engine with default parameters.

On the other hand, site-directed molecular docking was performed with Metascreener to evaluate the best docking pose for compound SE thoroughly. In this sense, the grid box of the docking simulations between SE and NS2B/NS3 was centered on the Cα of ASP75, which belongs to the catalytic triad of this protease, while the grid box of the docking between SE and NS5 was located in the Cα of VAL132 since it represents a residue essential for the enzymatic activity. Additional docking calculations were performed between these targets and the molecules from the database constructed as explained in section 10, which showed the highest similarities with SE (CHEMBL604091, CHEMBL1324, CHEMBL597978, and CHEMBL599409) to compare their interactions with the proteases.

### 4.13 Molecular Dynamics

Molecular Dynamics (MD) simulations for compound SE and the most similar known inhibitors in complex with NS5 and NS3 proteins were carried out to confirm their interaction with DENV proteases in a water-based system and at the time. MD simulations were performed at 100 ns for each complex. First, the topology of the ligand is generated using an automatic script that utilizes ACPYPE.^51,52^ The several steps of the MD simulations were done using GROMACS 2022.3.^53^ These simulations were launched on the Picasso supercomputer at the Universidad de Málaga (https://www.scbi.uma.es/site/) using GPU (NVIDIA A100-SXM4–40GB) and 4 GB of RAM. We first created the protein topology using the gmx pdb 2gmx command, specifying AMBER99SB as the force field.^54^ We defined the simulation box, solvated, and added ions. The next step was an energy minimization stage of 2000 ps. After that, a single NvT equilibration stage of 50000 ps and 5 NpT equilibration stages of 50,000 ps each were carried out. Finally, the dynamics were run at 200 ns, and the final trajectory generated was extracted in different frames to compare the ligands’ stability and movement regarding the binding pose. Finally, the trajectories are analyzed using the ASGARD tool (https://github.com/bio-hpc/ASGARD) to obtain the MM-PBSA analysis and the nonbonded interactions. In addition, several analyses were performed to validate the binding between the compounds and the target proteins. These included root-mean-square deviation (RMSD), RMSD fluctuation, radius of gyration (Rg), and hydrogen bond interaction analyses.

## Supplementary Material



## Notes

Spectra and HPLC of desacyl grazielia acid tiglate (Figures S1–S8). Anti-DENV activity and *in silico* assays (Figures S9–S23 and Table S1) (PDF)

## Abbreviations

DENV: dengue virus
EC_50_: concentration required to reduce virus yield by 50%
CC_50_: compound concentration necessary to reduce the MTT signal by 50% compared to cell controls treated with the vehicle
SI: selectivity index, calculated as
CC_50_/EC_50_; NS: dengue virus nonstructural proteins
STL: sesquiterpene lactone
C-STL: carboxylated-sesquiterpene lactone
compound SE: phytochemical isolated from *Stevia entreriensis* identified as desacyl grazielia acid tiglate
DMSO: dimethyl sulfoxide
TLC: thin layer chromatography
ANIS: anisaldehyde sulfuric
BG: bromocresol green
E protein: dengue virus envelope protein
