# Supplementary material for: A Promising Anti-dengue Virus Carboxylated-Sesquiterpene Lactone from Stevia entreriensis (Asteraceae)
Source: ACS Omega. 2025 Oct 9;10(41):48228–41. doi: 10.1021/acsomega.5c05127 (PMC12547525; doi:10.1021/acsomega.5c05127)
Supplement: Supplementary file 1 [file ao5c05127_si_001.pdf]

Supporting information

A promising anti-dengue virus carboxylated-sesquiterpene lactone from *Stevia entreriensis* (Asteraceae)

Jimena Borgo[1,†], Alejandro Rodríguez-Martínez[2,3,†], Mariel S. Wagner[4], Augusto E. Bivona[5], Claudia S. Sepúlveda[6], César A.N. Catalán[7], Horacio Pérez-Sánchez[2,‡,], Valeria P. Sülsen[1,‡,*]*

[1] Instituto de Química y Metabolismo del Fármaco, (IQUIMEFA) CONICET-Universidad de Buenos Aires; Cátedra de Farmacognosia, Facultad de Farmacia y Bioquímica, Universidad de Buenos Aires, Buenos Aires C1113AAD, Argentina.

[2] Structural Bioinformatics and High Performance Computing Research Group (BIO-HPC), HiTech Innovation Hub, Universidad Católica San Antonio de Murcia (UCAM), Murcia 30107, Spain.

[3] Health Sciences PhD Program, Universidad Católica de Murcia (UCAM), Murcia, 30107, Spain

[4] Laboratorio de Estrategias Antivirales, Departamento de Química Biológica, Facultad de Ciencias Exactas y Naturales, Universidad de Buenos Aires, Buenos Aires C1428EGA, Argentina.

[5] Instituto de Estudios de la Inmunidad Humoral Prof. Ricardo A. Margni (IDEHU), (Universidad de Buenos Aires- CONICET), Buenos Aires C1113AAD, Argentina; Cátedra de Inmunología, Facultad de Farmacia y Bioquímica, Universidad de Buenos Aires, Buenos Aires, C1113AAD, Argentina.

[6] Instituto de Química Biológica de la Facultad de Ciencias Exactas y Naturales (IQUIBICEN), CONICET-Universidad de Buenos Aires, Buenos Aires C1428EGA, Argentina.

[7] Instituto de Química Orgánica, Facultad de Bioquímica, Química y Farmacia, Universidad Nacional de Tucumán, San Miguel de Tucumán T4000INI, Tucumán, Argentina.

*Corresponding authors: hperez@ucam.edu, vsulsen@ffyb.uba.ar

† These authors contributed equally to this work.

‡ The work was co-directed by both authors.

Table of content

Figure S1. HRESIMS of the isolated compound from *Stevia entreriensis*

Figure S2. IR spectrum of desacyl grazielia acid tiglate isolated from *Stevia entreriensis*

Figure S3. ^{1}H-NMR spectrum of desacyl grazielia acid tiglate isolated from *Stevia entreriensis* (600 MHz, $CDCl_3$).

Figure S4. ^{13}C-NMR spectrum of desacyl grazielia acid tiglate isolated from *Stevia entreriensis* (125 MHz, $CDCl_3$).

Figure S5. ^{1}H-^{1}H COSY spectrum of desacyl grazielia acid tiglate isolated from *Stevia entreriensis* (600 MHz, $CDCl_3$).

Figure S6. HSQC spectrum of desacyl grazielia acid tiglate isolated from *Stevia entreriensis* (600 MHz, $CDCl_3$).

Figure S7. HMBC spectrum of desacyl grazielia acid tiglate isolated from *Stevia entreriensis* (600 MHz, $CDCl_3$).

Figure S8. Purity assessment of desacyl grazielia acid tiglate isolated from *Stevia entreriensis.*

Figure S9. Anti-DENV-2 activity of desacyl grazielia acid tiglate.

Figure S10. Electrostatic grid of desacyl grazielia acid tiglate against molecules CHEMBL604091, CHEM1324, CHEMBL597978, and CHEMBL 599409.

Figure S11A. 3D representations of the compounds aligned against the pharmacophore model.

Figure S11B. 2D structures of the compounds, highlighting the matching features for each one.

Figure S12A. RMSD representations of the NS2B/NS3- desacyl grazielia acid tiglate complex

Figure S12B. RMSD representations of the NS2B/NS3-CHEMBL1324 complex.

Figure S13A. RMSD representations of NS5 complexes for the NS5- desacyl grazielia acid tiglate complex.

Figure S13B. RMSD representations of the NS5-CHEMBL597978 complex

Figure S13C. RMSD representations of the NS5-CHEMBL599409 complex

Figure S13D. RMSD representations of the NS5-CHEMBL604091 complex.

Figure S14A. RMSF representations of the NS2B/NS3 target for the NS2B/NS3- desacyl grazielia acid tiglate complex.

Figure S14B. RMSF representations of the desacyl grazielia acid tiglate for the NS2B/NS3- desacyl grazielia acid tiglate complex.

Figure S15A. RMSF representations of the NS2B/NS3 target for the NS2B/NS3-CHEMBL1324 complex.

Figure S15B. RMSF representations of the CHEMBL1324 for the NS2B/NS3-CHEMBL1324 complex.

Figure S16A. RMSF representations of the NS5 target for the RMSD representations of the NS5- desacyl grazielia acid tiglate complex.

Figure S16B. RMSF representations of the desacyl grazielia acid tiglate for the RMSD representations of the NS5-desacyl grazielia acid tiglate complex.

Figure S17A. RMSF representations of the NS5 target for the NS5- CHEMBL597978 complex.

Figure S17B. RMSF representations of the CHEMBL597978 for the NS5- CHEMBL597978 complex.

Figure S18A. RMSF representations of the NS5 target for the NS5- CHEMBL599409 complex.

Figure S18B. RMSF representations of the CHEMBL599409 for the NS5- CHEMBL599409 complex.

Figure S19A. RMSF representations of the NS5 target for the NS5- CHEMBL604091 complex.

Figure S19B. RMSF representations of the CHEMBL604091 for the NS5- CHEMBL604091 complex.

Figure S20A. Radius of gyration (Rg) representation of the NS2B/NS3- desacyl grazielia acid tiglate complex

Figure S20B. Radius of gyration (Rg) representation of the NS2B/NS3-CHEMBL1324 complex.

Figure S21A. Radius of gyration (Rg) representation of the NS5- desacyl grazielia acid tiglate complex.

Figure S21B. Radius of gyration (Rg) representation of the NS5-CHEMBL597978 complex

Figure S21C. Radius of gyration (Rg) representation of the NS5-CHEMBL599409 complex

Figure S21D. Radius of gyration (Rg) representation of the NS5-CHEMBL604091 complex.

Figure S22A. Hydrogen Bonds analysis of the NS2B/NS3- desacyl grazielia acid tiglate complex

Figure S22B. Hydrogen Bonds analysis of the NS2B/NS3-CHEMBL1324 complex.

Figure S23A. Hydrogen Bonds analysis of the NS5- desacyl grazielia acid tiglate complex.

Figure S23B. Hydrogen Bonds analysis of the NS5-CHEMBL597978 complex

Figure S23C. Hydrogen Bonds analysis of the NS5-CHEMBL599409 complex

Figure S23D. Hydrogen Bonds analysis of the NS5-CHEMBL604091 complex.

Table S1. Blind docking results for desacyl grazielia acid tiglate with the different DENV-2 targets.

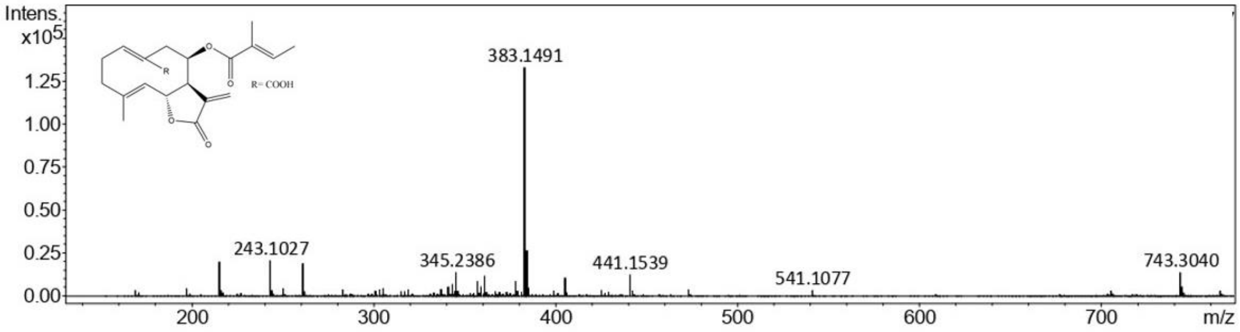

Figure S1. HRESIMS of the isolated compound from *Stevia entreriensis*.

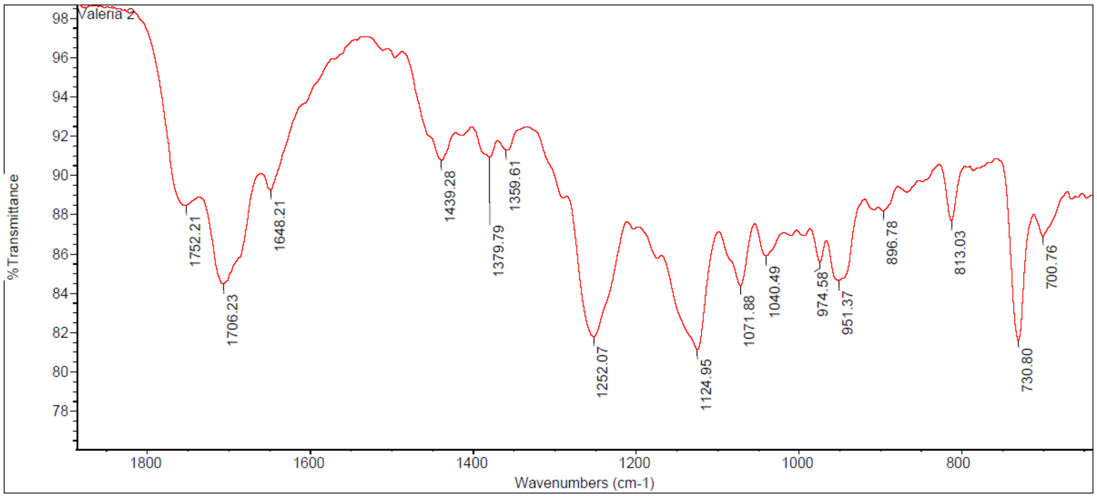

Figure S2. IR spectrum of of desacyl grazielia acid tiglate isolated from *Stevia entreriensis*.

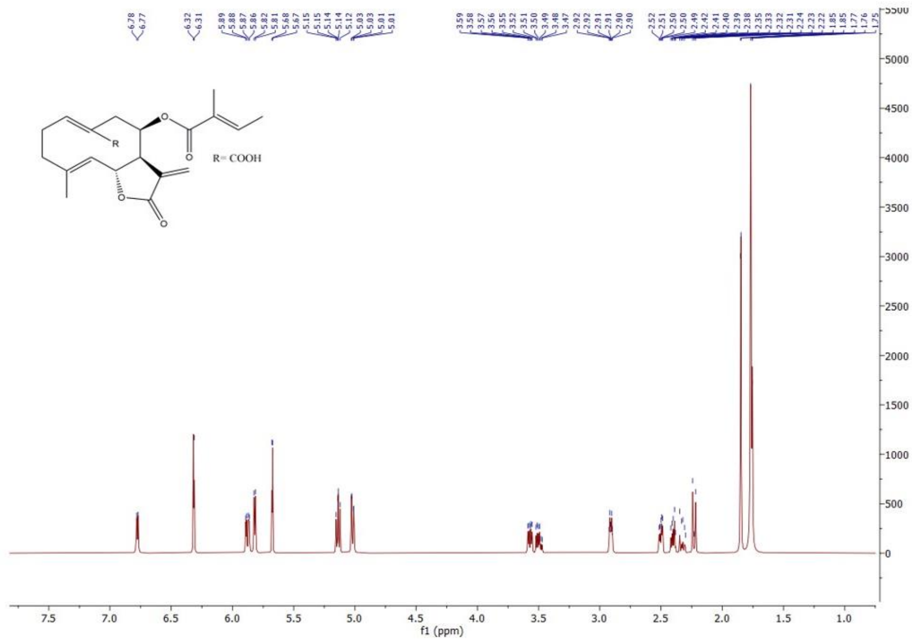

Figure S3. ^{1}H-NMR spectrum of desacyl grazielia acid tiglate isolated from *Stevia entreriensis* (600 MHz, $CDCl_3$).

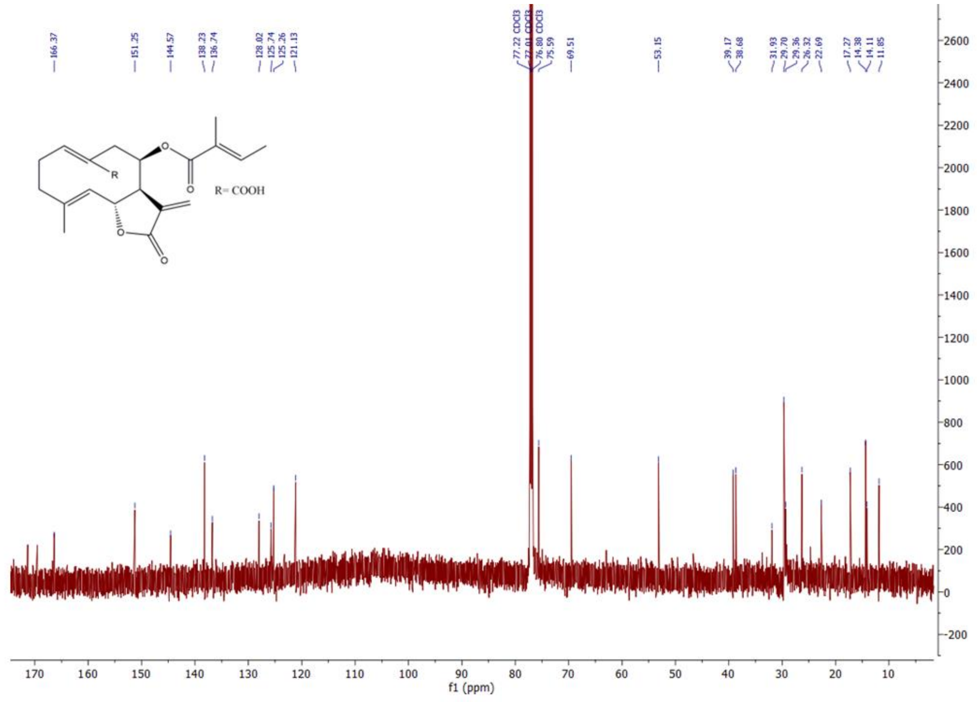

Figure S4. ^{13}C-NMR spectrum of desacyl grazielia acid tiglate isolated from *Stevia entreriensis* (125 MHz, $CDCl_3$).

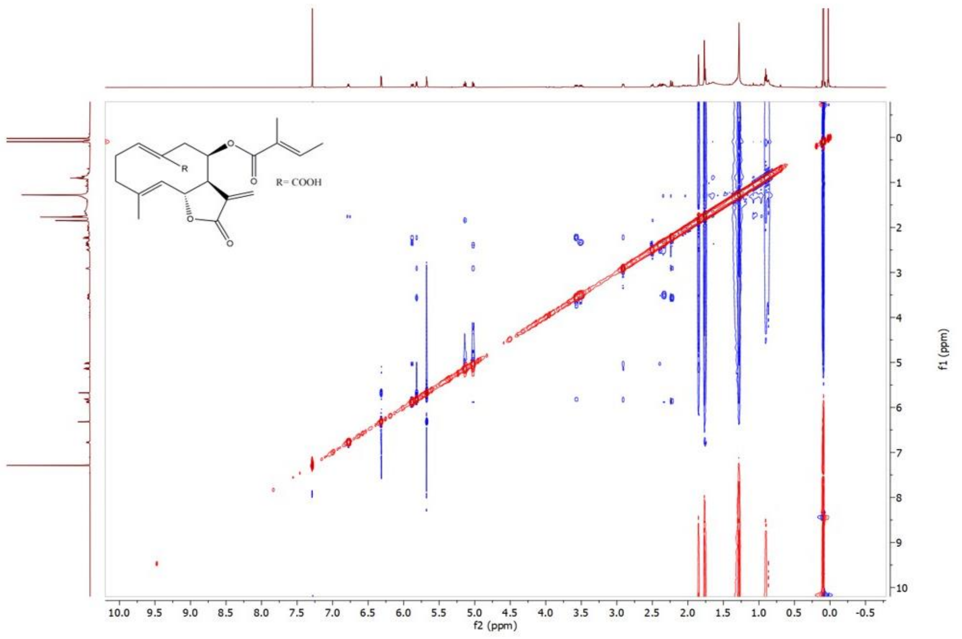

Figure S5. ^{1}H-^{1}H COSY spectrum of desacyl grazielia acid tiglate isolated from *Stevia entreriensis* (600 MHz, $CDCl_3$).

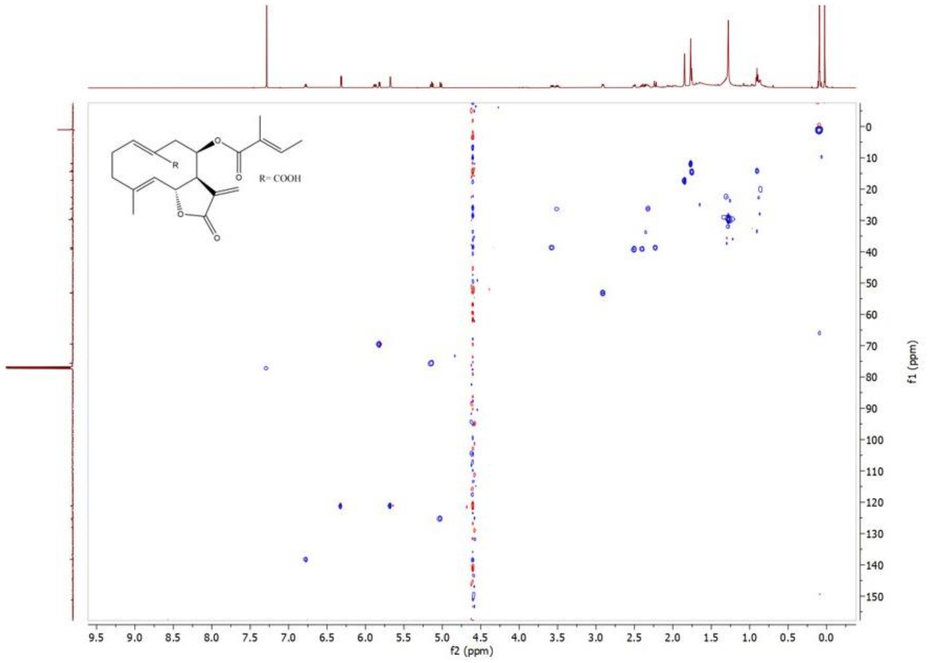

Figure S6. HSQC spectrum of desacyl grazielia acid tiglate isolated from *Stevia entreriensis* (600 MHz, $CDCl_3$).

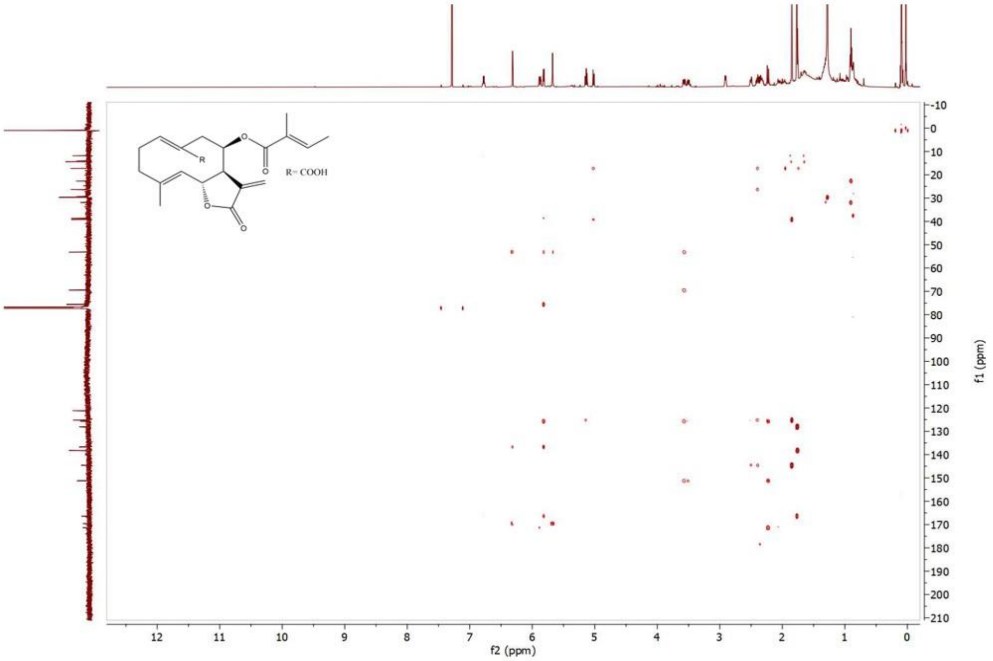

Figure S7. HMBC spectrum of desacyl grazielia acid tiglate isolated from *Stevia entreriensis* (600 MHz, $CDCl_3$).

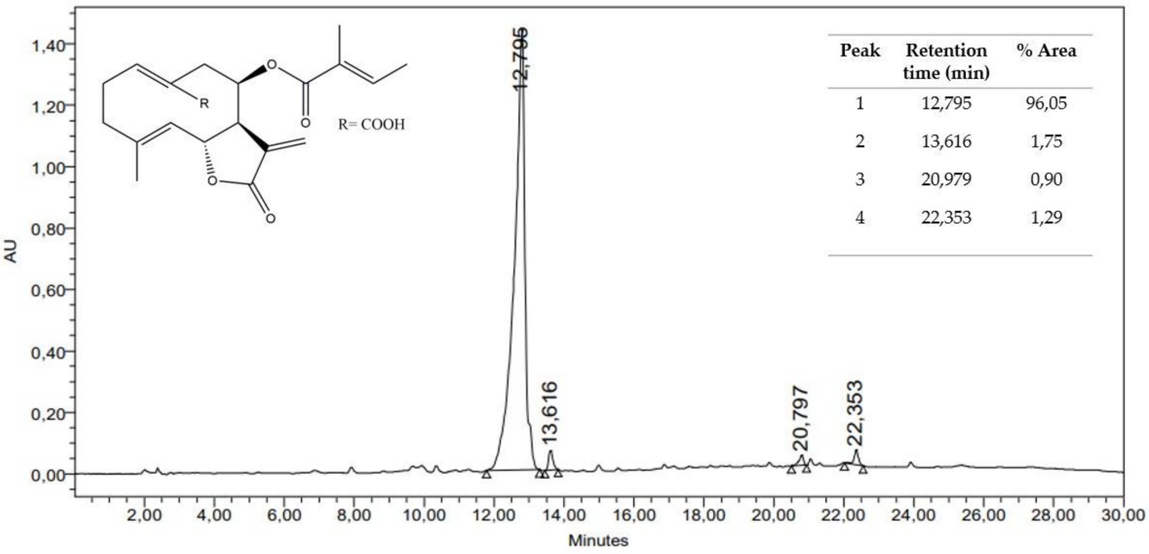

Figure S8. Purity assessment of desacyl grazielia acid tiglate isolated from *Stevia entreriensis*

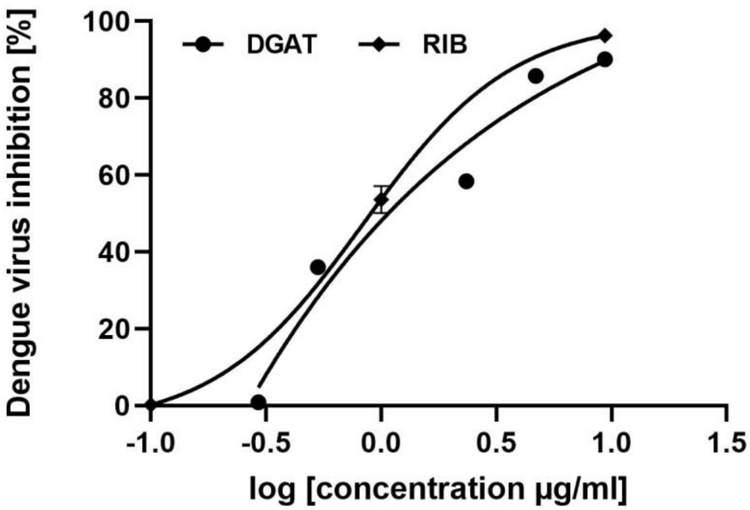

Figure S9. Anti-DENV-2 activity of desacyl grazielia acid tiglate (DGAT) and rivabirin (RIB). Inhibition values (% respect to non-treated viral control) are presented as the mean ± SD obtained from three independent experiments.

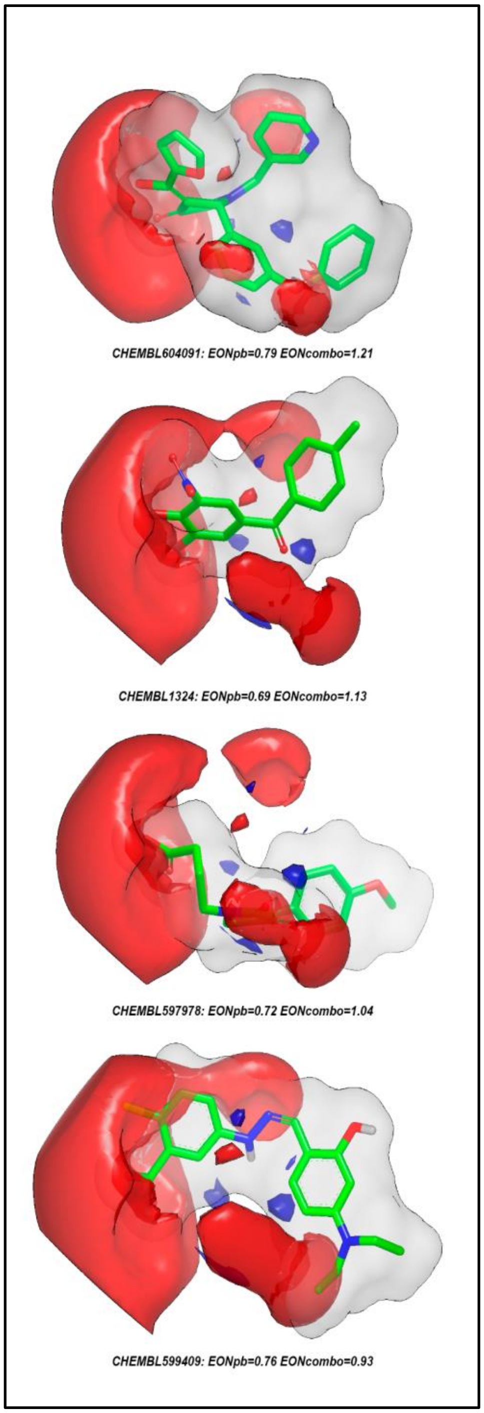

Figure S10. Electrostatic grid of desacyl grazielia acid tiglate against molecules CHEMBL604091, CHEM1324, CHEMBL597978, and CHEMBL 599409.

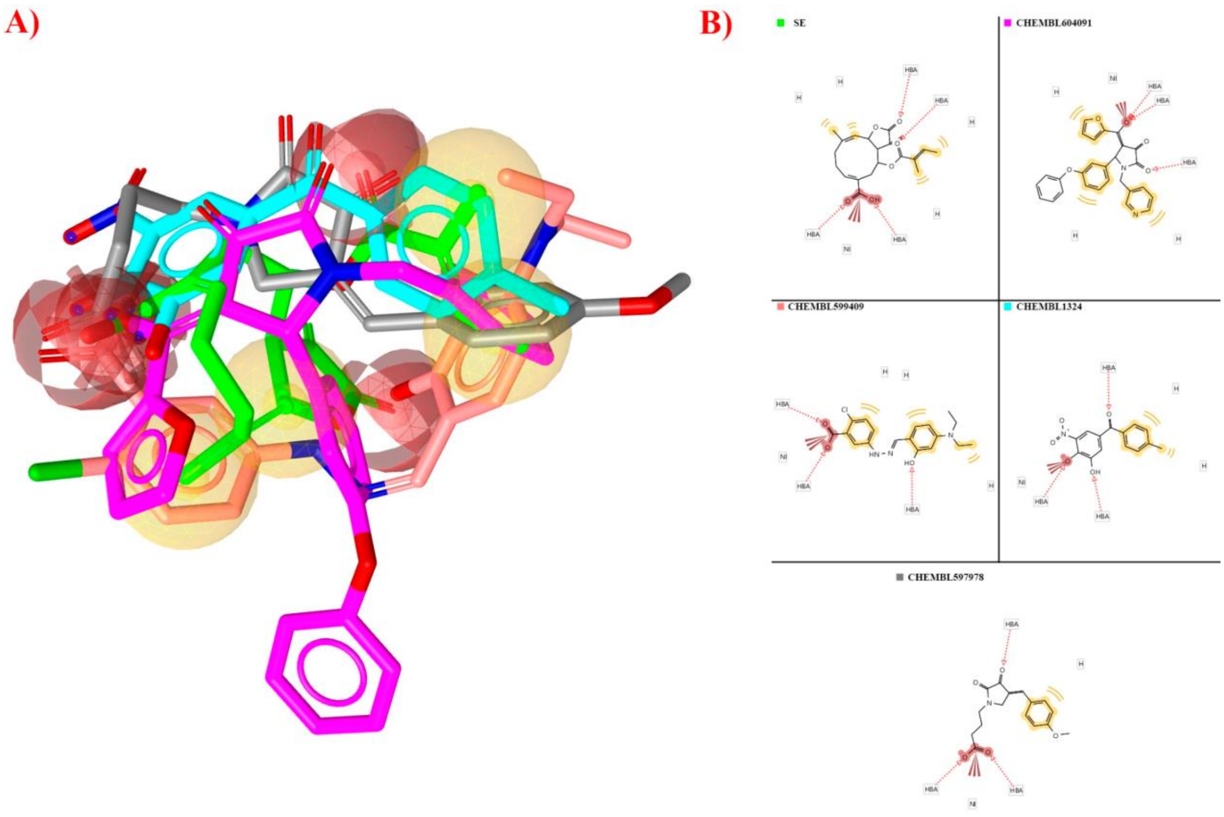

Figure S11. A) 3D diagram of the SE pharmacophoric features against SE (green), CHEMBL604091 (magenta), CHEMBL599409 (pink), CHEMBL1324 (cyan), and CHEMBL597978 (gray). **B)** 2D diagrams of the matching features for each molecule

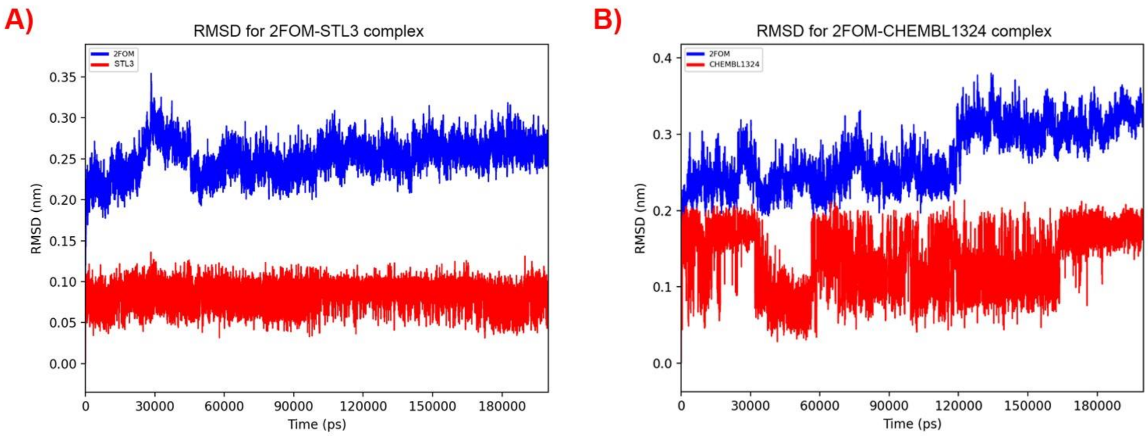

Figure S12. A) RMSD representations of the NS2B/NS3- desacyl grazielia acid tiglate complex **B)** RMSD representations of the NS2B/NS3-CHEMBL1324 complex.

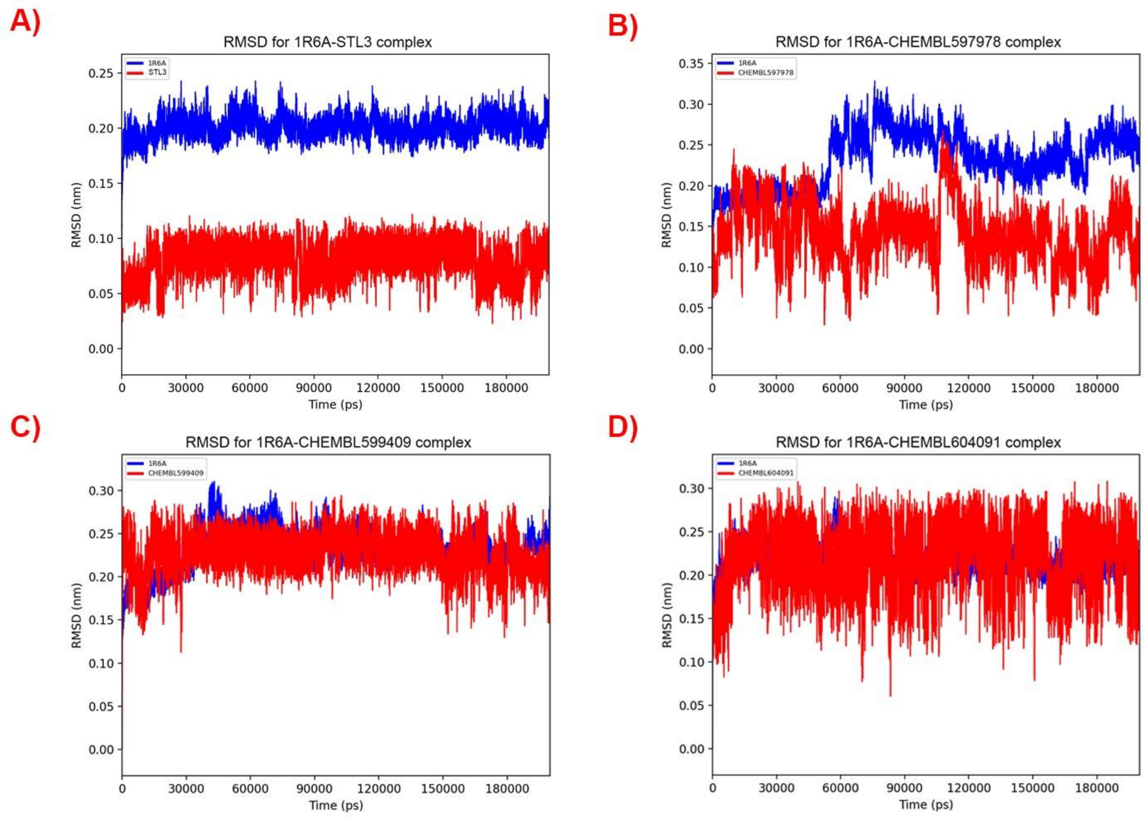

Figure S13. A). RMSD representations of NS5 complexes for the NS5- desacyl grazielia acid tiglate complex **B)**. RMSD representations of the NS5-CHEMBL597978 complex. **C)**. RMSD representations of the NS5-CHEMBL599409 complex**. Figure D)**. RMSD representations of the NS5-CHEMBL604091 complex.

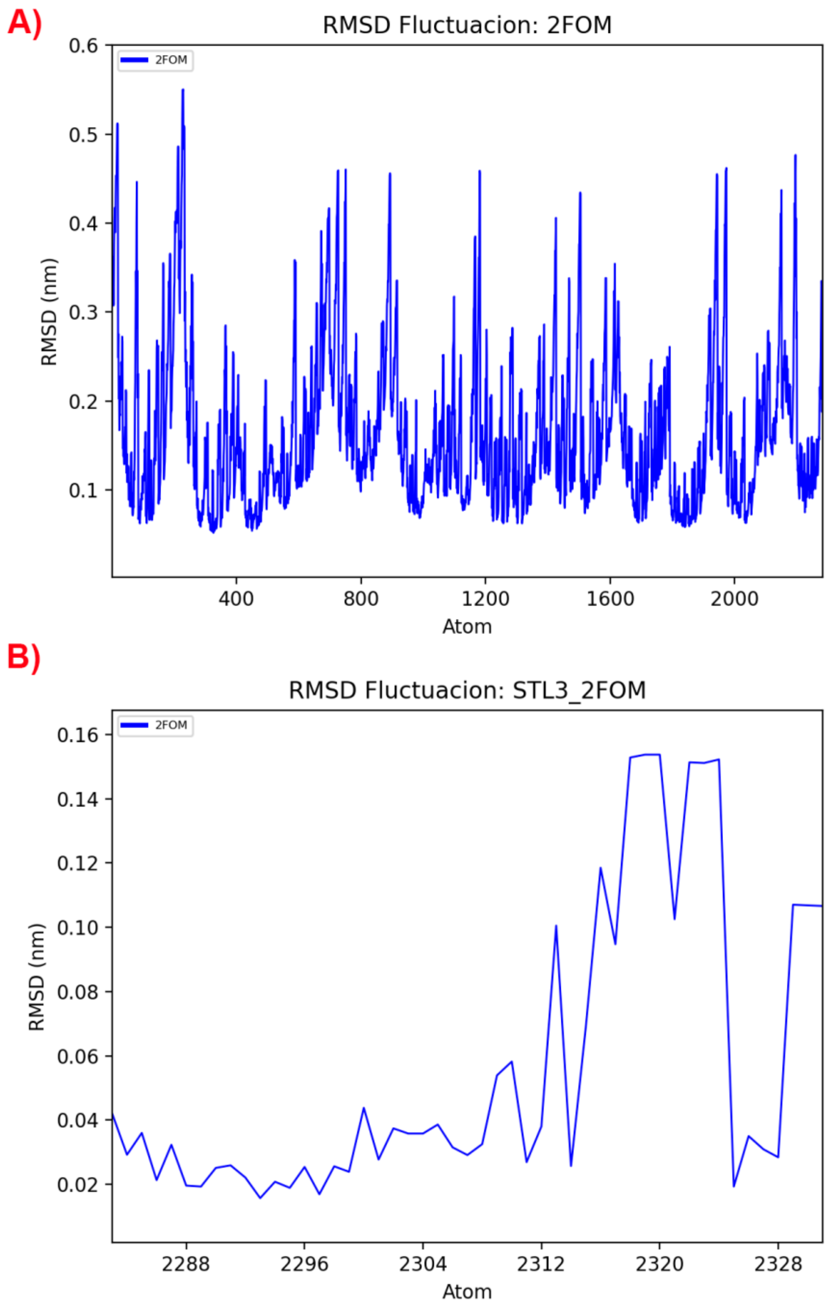

Figure S14. **A**) RMSF representations of the NS2B/NS3- desacyl grazielia acid tiglate complex. **A)** RMSF representation for NS2B/NS3 target. **B)** RMSF representation for desacyl grazielia acid tiglate.

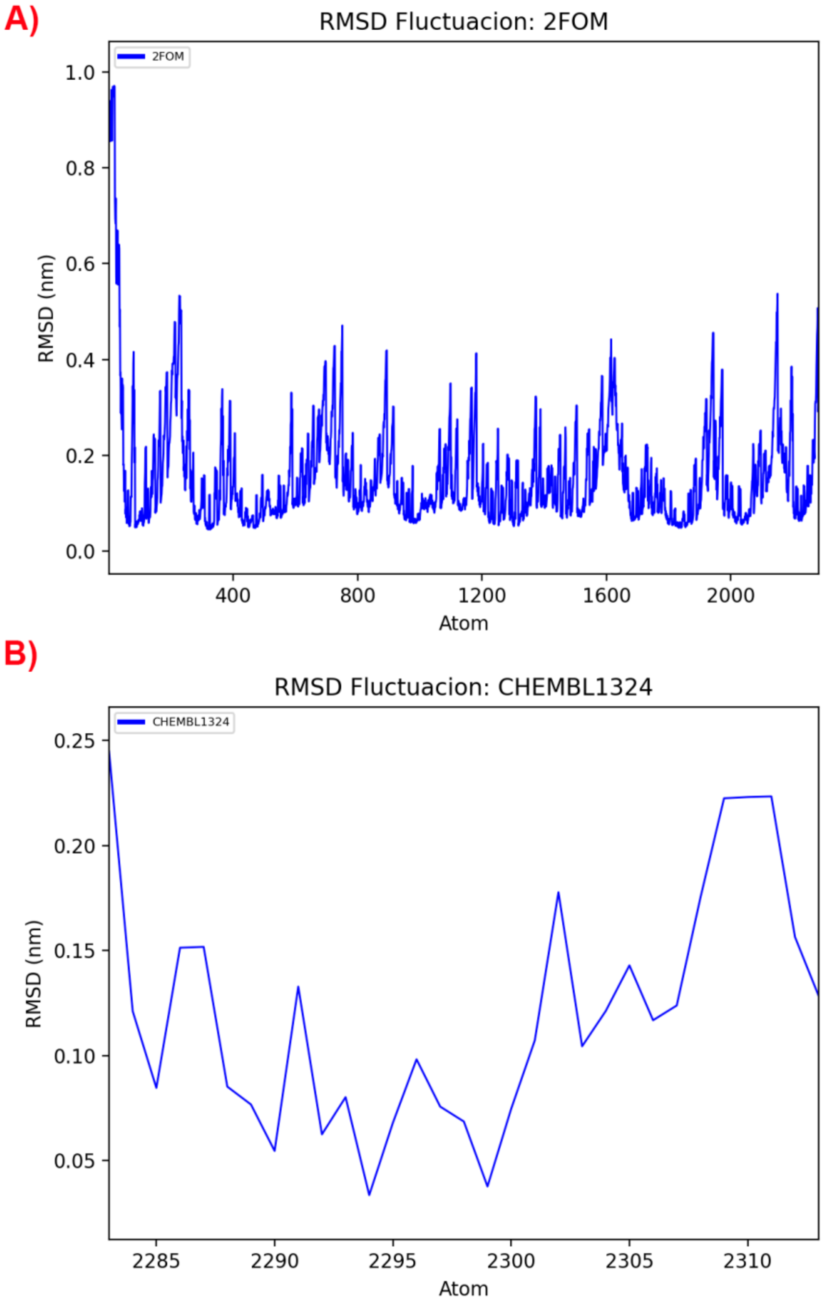

Figure S15. RMSF representations of the RMSD representations of the NS2B/NS3-CHEMBL1324 complex. **A)** RMSF representation for NS2B/NS3 target. **B)** RMSF representation for CHEMBL1324.

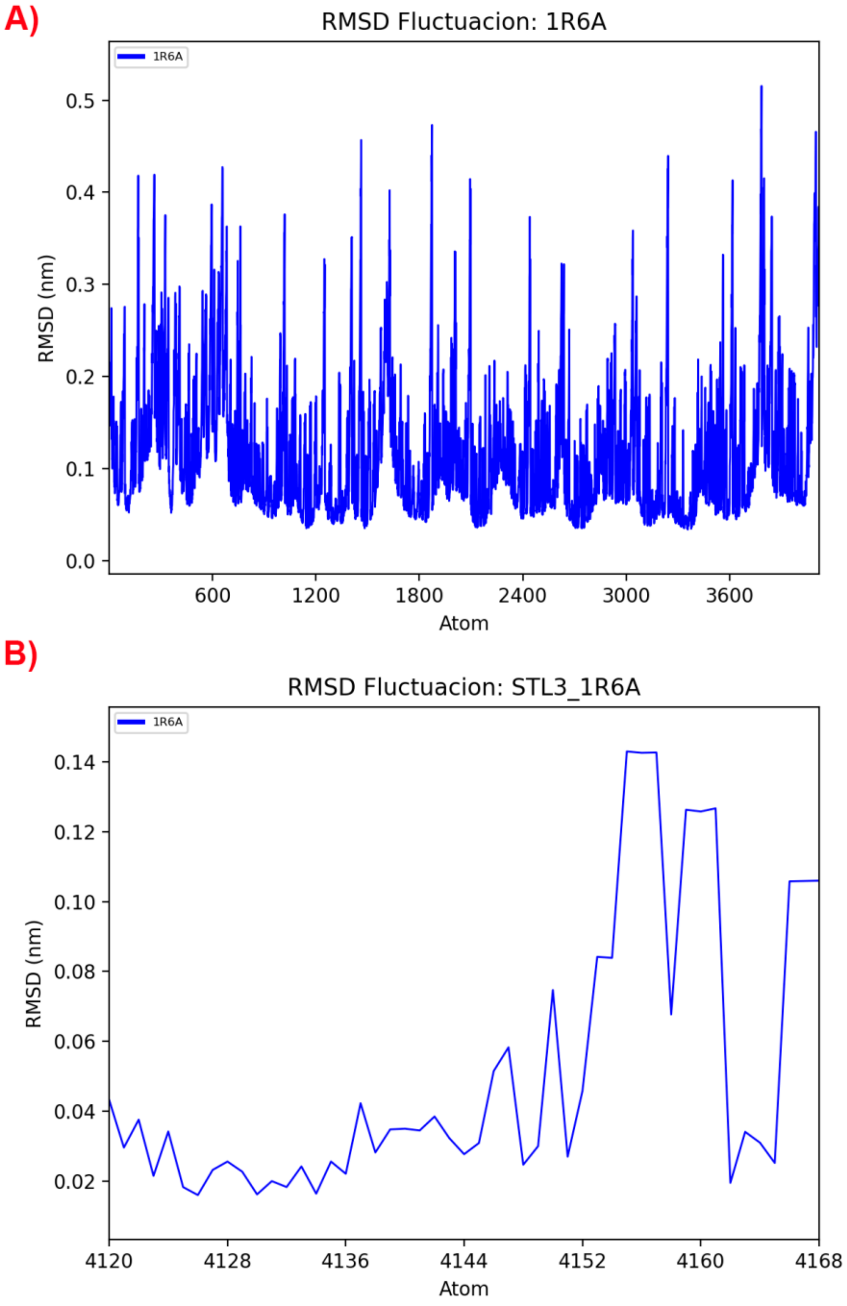

Figure S16. RMSF representations of the RMSD representations of the NS5-CHEMBL1324 complex. **A)** RMSF representation for NS5 target. **B)** RMSF representation for desacyl grazielia acid tiglate.

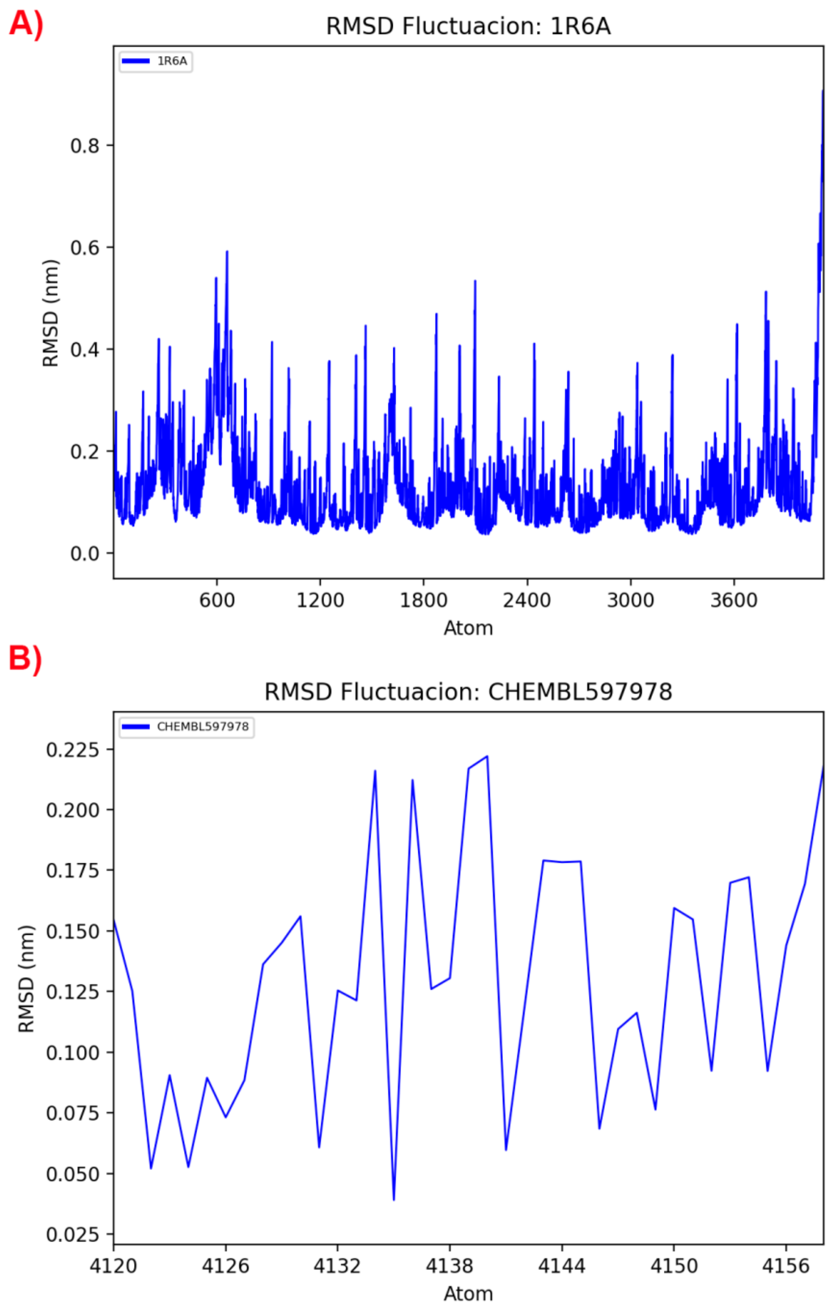

Figure S17. RMSF representations of the RMSD representations of the NS5- CHEMBL597978 complex. **A)** RMSF representation for NS5 target. **B)** RMSF representation for CHEMBL597978.

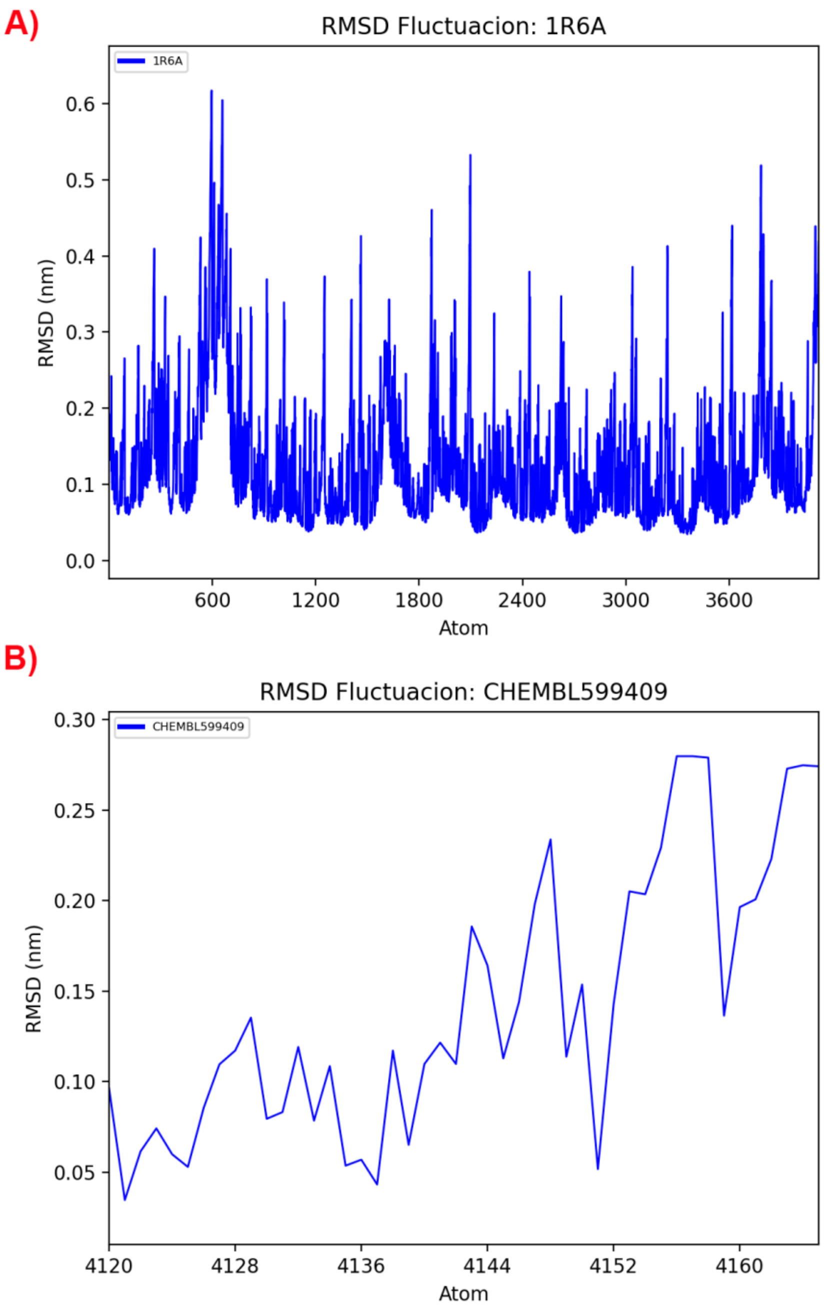

Figure S18. RMSF representations of the RMSD representations of the NS5- CHEMBL599409 complex. **A)** RMSF representation for NS5 target. **B)** RMSF representation for CHEMBL599409.

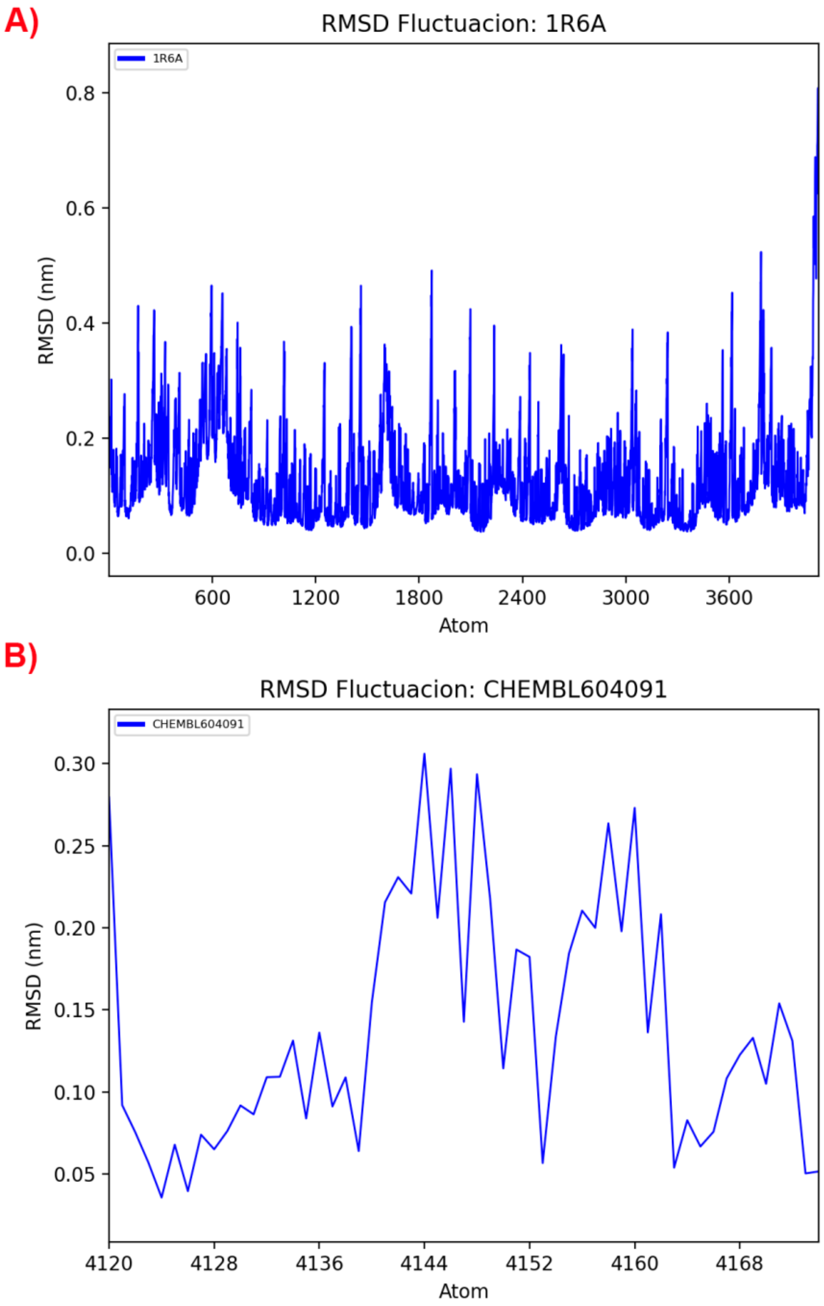

Figure S19. RMSF representations of the RMSD representations of the NS5- CHEMBL604091 complex. **A)** RMSF representation for NS5 target. **B)** RMSF representation for CHEMBL604091.

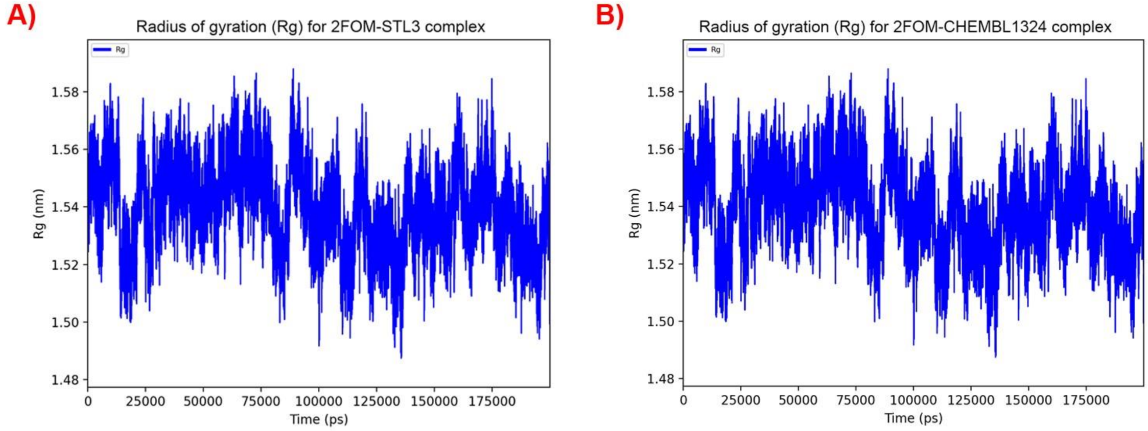

Figure S20. A) Radius of gyration (Rg) representations of the NS2B/NS3- desacyl grazielia acid tiglate complex **B)** RMSD representations of the NS2B/NS3-CHEMBL1324 complex.

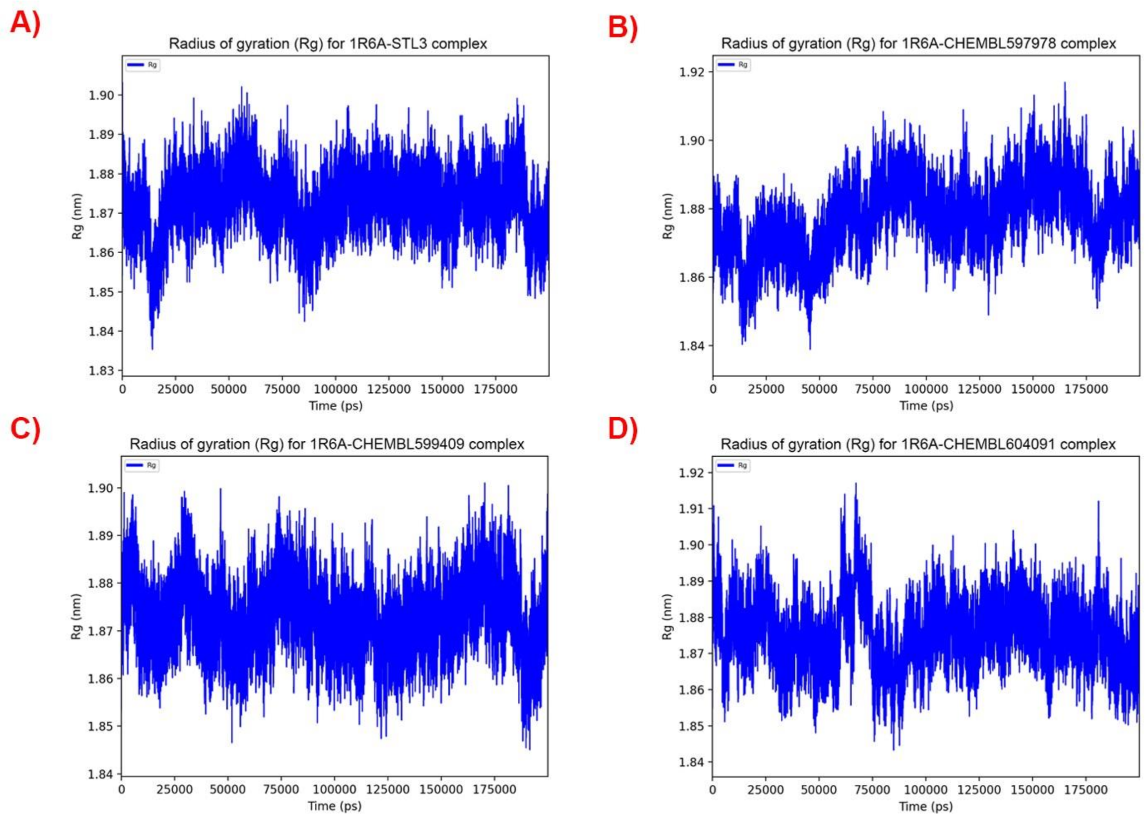

Figure S21. A). Radius of gyration (Rg) representation of NS5 complexes for the NS5- desacyl grazielia acid tiglate complex. **B)**. Radius of gyration (Rg) representation of the NS5-CHEMBL597978 complex. **C)**. Radius of gyration (Rg) representation of the NS5-CHEMBL599409 complex**. Figure D)**. Radius of gyration (Rg) representation of the NS5-CHEMBL604091 complex.

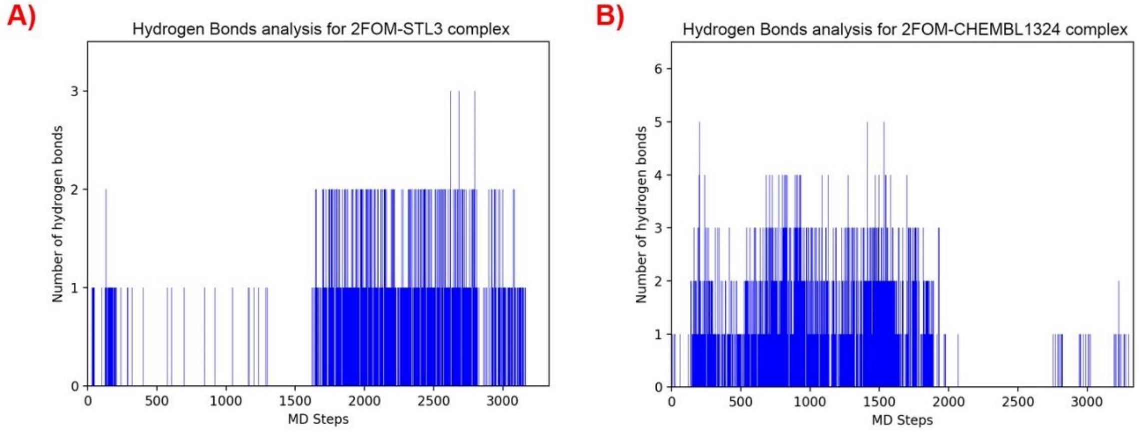

Figure S22. A) Hydrogen Bonds analysis of the NS2B/NS3- desacyl grazielia acid tiglate complex **B)** Hydrogen Bonds analysis of the NS2B/NS3-CHEMBL1324 complex.

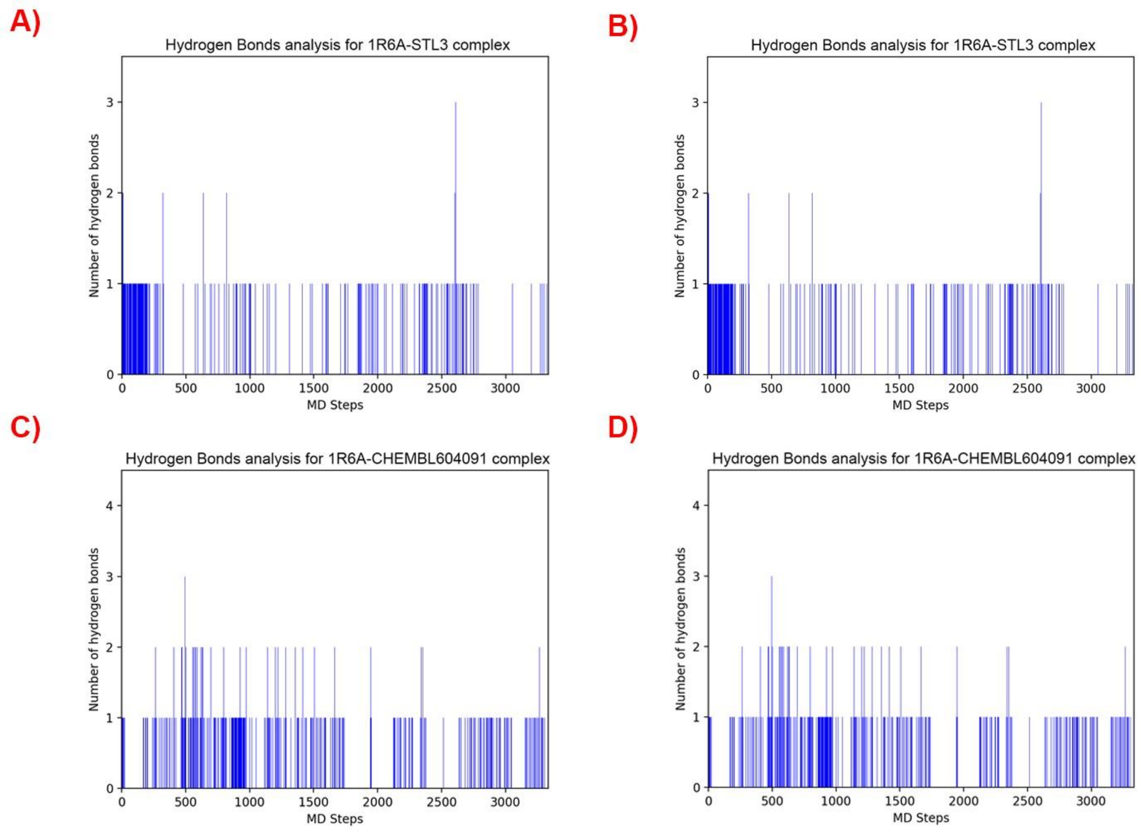

Figure S23. A). Hydrogen Bonds analysis of NS5 complexes for the NS5- desacyl grazielia acid tiglate complex. **B)**. Hydrogen Bonds analysis of the NS5-CHEMBL597978 complex. **C)**. Hydrogen Bonds analysis of the NS5-CHEMBL599409 complex**. D)**. Hydrogen Bonds analysis of the NS5-CHEMBL604091 complex.

Table S1. Blind docking results for desacyl grazielia acid tiglate **with the different DENV-2 targets**

NS1			NS2B/NS3				NS5				E protein		
Docking score (kcal/mol)	Hydrophobic interactions (Å)	Hydrogen bonds (Å)	Docking score (kcal/mol)	Hydrophobic interactions (Å)	Hydrogen bonds (Å)	Salt bridges (Å)	Docking score (kcal/mol)	Hydrophobic interactions (Å)	Hydrogen bonds (Å)	Salt bridges (Å)	Docking score (kcal/mol)	Hydrophobic interactions (Å)	Salt bridges (Å)
-5.4	ASN76 (3.88)	ASN130 (3.19)	-7.1	LEU128 (3.58)	SER135 (3.03)	HIS51 (4.92)	-8.2	THR104 (3.8)	GLU111 (3.18)	LYS105 (5.49)	-6.2	PRO243 (3.48	HIS244 (4.14)
	GLU83 (3.76)			LEU128 (3.88)	GLY153 (3.29)	HIS51 (4.39)		LYS105 (3.71)	GLY148 (3.05)	HIS110 (4.06)		THR268 (3.66)	
	THR87 (3.68)			TYR161 (4.00)				LYS105 (3.48)	GLU149 (3.04)			GLU269 (3.58)	
				TYR161 (3.68)				VAL132 (3.48)				LEU278 (3.67)	
				TYR161 (3.7)				VAL132 (3.72)				LEU278 (3.73)	
				TYR161 (3.86)				PHE133 (3.67)				PHE279 (3.70)	
								ILE147 (3.63)				PHE279 (3.71)	
								GLU149 (3.71)				PHE279 (3.71)	
												PHE279 (3.79)	
